# Evolutionary Dynamics of Mexican Lineage H5N2 Avian Influenza Viruses

**DOI:** 10.3390/v14050958

**Published:** 2022-05-03

**Authors:** Wanhong Xu, Roberto Navarro-López, Mario Solis-Hernandez, Francisco Liljehult-Fuentes, Miguel Molina-Montiel, María Lagunas-Ayala, Marisol Rocha-Martinez, Eduardo Ferrara-Tijera, Juan Pérez de la Rosa, Yohannes Berhane

**Affiliations:** 1 National Centre for Foreign Animal Disease, Winnipeg, MB R3E 3M4, Canada; wanhong.xu@inspection.gc.ca; 2 Animal Health General Directorate, Animal and Plant Health, Food Inspection and Food Safety National Services (SENASICA), Secretariat of Agriculture, Livestock, Rural Development, Fisheries and Food (SAGARPA), Mexico City 06470, Mexico; roberto.navarro@senasica.gob.mx (R.N.-L.); miguel.molina.i@senasica.gob.mx (M.M.-M.); maria.lagunas.i@senasica.gob.mx (M.L.-A.); marisol.rocha@senasica.gob.mx (M.R.-M.); eduardo.ferrara.i@senasica.gob.mx (E.F.-T.); juan.perez@senasica.gob.mx (J.P.d.l.R.); 3 United States-Mexico Commission for the Prevention of Foot-and-Mouth Disease and Other Exotic Diseases of Animals, Mexico City 64590, Mexico; mario.solis@senasica.gob.mx (M.S.-H.); francisco.liljehult@senasica.gob.mx (F.L.-F.); 4 Department of Animal Science, University of Manitoba, Winnipeg, MB R3T 2S2, Canada

**Keywords:** H5N2 avian influenza virus, antigenic evolution, selection pressure, genetic and phylogenetic analyses

## Abstract

We have demonstrated for the first time a comprehensive evolutionary analysis of the Mexican lineage H5N2 avian influenza virus (AIV) using complete genome sequences (*n* = 189), from its first isolation in 1993 until 2019. Our study showed that the Mexican lineage H5N2 AIV originated from the North American wild bird gene pool viruses around 1990 and is currently circulating in poultry populations of Mexico, the Dominican Republic, and Taiwan. Since the implementation of vaccination in 1995, the highly pathogenic AIV (HPAIV) H5N2 virus was eradicated from Mexican poultry in mid-1995. However, the low pathogenic AIV (LPAIV) H5N2 virus has continued to circulate in domestic poultry populations in Mexico, eventually evolving into five distinct clades. In the current study, we demonstrate that the evolution of Mexican lineage H5N2 AIVs involves gene reassortments and mutations gained over time. The current circulating Mexican lineage H5N2 AIVs are classified as LPAIV based on the amino acid sequences of the hemagglutinin (HA) protein cleavage site motif as well as the results of the intravenous pathogenicity index (IVPI). The immune pressure from vaccinations most likely has played a significant role in the positive selection of antigenic drift mutants within the Mexican H5N2 AIVs. Most of the identified substitutions in these viruses are located on the critical antigenic residues of the HA protein and as a result, might have contributed to vaccine failures. This study highlights and stresses the need for vaccine updates while emphasizing the importance of continued molecular monitoring of the HA protein for its antigenic changes compared to the vaccines used.

## 1. Introduction

Avian influenza viruses (AIVs) are classified in the family *Orthomyxoviridae*, genus *Influenzavirus A*, and have negative-sense RNA genomes consisting of eight segments coding for at least 10 proteins [1,2]. To date, 16 hemagglutinin (HA) and nine neuraminidase (NA) subtypes of AIVs have been isolated from aquatic and domestic birds [3]. According to genetic features and/or disease severity in chickens, AIVs can also be classified into low pathogenic (LP) and highly pathogenic (HP) pathotypes [4]. Wild aquatic birds are the main reservoir of AIVs, and in these species, infections are generally non-pathogenic. Spillover of AIVs from wild aquatic birds to poultry and other avian species occurs frequently. Many of these spillover infections are transient; however, these viruses may adapt to their new avian hosts through the acquisition of mutations [5]. Infections with AIVs of H5 and H7 subtypes are of greatest concern following transmission to poultry because of their potential to evolve into highly pathogenic forms that can be devastating to domestic poultry populations and occasionally can be transmitted to humans [5].

The first evidence of the presence of H5N2 AIV in Mexican poultry was demonstrated serologically in October 1993 [6], and the first isolate was A/chicken/Mexico/31381–7/93 [7]. The following year in May 1994, a mildly pathogenic H5N2 virus was isolated from chickens (A/chicken/Mexico/26654–1374/94). As this H5N2 virus was widely spread in poultry populations, mass culling was not implemented for eradication. Within several months in the late fall of 1994, a moderately pathogenic H5N2 virus (A/chicken/Puebla/8624–602/94) that killed four out of eight chickens inoculated intravenously was identified. Later, a highly pathogenic H5N2 virus (A/chicken/Queretaro/14588–19/95) was isolated in the early winter of 1995 [7]. Vaccination was implemented to control the spread of the HPAI virus in 1995 using an inactivated vaccine based on A/chicken/Mexico/CPA-232/94 (H5N2) and soon after, eradication of the H5N2 HPAI viruses was accomplished in mid-1995, leading Mexico to announce a declaration of freedom from H5N2 HPAI in December 1995. Since then, vaccination continues to be used to protect commercial flocks from low pathogenic H5N2 AIV outbreaks. In addition to the inactivated vaccine, Mexico began to use recombinant vector vaccines in 1998. Despite the administration of billions of doses of inactivated and recombinant vector vaccines, H5N2 LPAIVs continued to circulate in domestic poultry flocks. To mitigate vaccine failures, efforts have been made to match vaccine seeds that can provide potent immune responses against circulating AIV strains. For example, inactivated vaccine seed strains of H5N2 AIV were changed twice because of the lack of protection offered by existing vaccines. The inactivated vaccine formulated from one of the earlier strains called A/chicken/Mexico/CPA-232/94 was replaced by A/chicken/Mexico/Vacuna CPA/2005 in 2009, and recently, A/chicken/Mexico/Vacuna CPA/2005 was replaced by A/chicken/Guanajuato/CPA-20966–15-VS/2015. Moreover, a commercially available recombinant Newcastle disease virus (NDV) expressing the H5 gene of A/chicken/Mexico/435/2005 has been administered to poultry since 1998 [8].

Despite vaccination efforts, repeated AIV surveillance, and biosecurity measures, H5N2 LPAIVs remain enzootic in poultry populations in Mexico. This LP virus has spread to neighboring countries of Guatemala in 1998 and EI Salvador in 2001 [9] and emerged in chickens in 2003 in Taiwan [10], 2005 in Japan [11], and in 2007 in the Dominican Republic [12]. The Mexican lineage H5N2 virus has been circulating in the poultry populations for over two decades. However, global-scale descriptions of its origins and evolutionary dynamics are lacking. To fill this gap, we inferred important aspects of Mexican lineage H5N2 AIV evolution such as the dynamics of global growth of the effective population size, the approximate time of origin, the evolution rate, the positive selection, and the amino acid mutations that might have played a role in the antigenic change. To this end, we performed a phylogenetic analysis by combining all publicly available complete Mexican lineage H5N2 AIV genomes (*n* = 83) and 106 new complete full genomes generated in this study using both maximum likelihood (ML) and Bayesian approaches. Furthermore, to determine H5 HA genetic diversity, cleavage site motif, and antigenic evolution, we performed the analysis of 284 complete HA genes related to the Mexican lineage H5N2 virus.

## 2. Materials and Methods

### 2.1. Virus Isolation and Genome Sequencing

A total of 106 samples sequenced in this study were derived from H5N2 AIVs that were isolated from Mexican poultry (Supplementary Table S1). These samples were processed at the animal health diagnostic laboratories of the Animal Health General Directorate, Animal & Plant Health, Food Inspection, and Food Safety National Services (SENASICA), Mexico. All the procedures for AIV isolation in chicken embryonated eggs were approved by the Care and Use of Animals Committee of the SENASICA based on the guidelines established by the Official Mexican Standard NOM-062-ZOO-1999 and the Internal Committee for the Care and Use of Laboratory Animals (CICUAL) belonging to the CPA-SENASICA (authorization number CICUAL-CPA-002-2022). Viral RNA extraction was performed using the High Pure RNA isolation kit (Roche Diagnostics, Laval, QC, Canada) according to the manufacturer’s protocol. The entire genome (PB2, PB1, PA, HA, NP, NA, M, and NS) of each virus was amplified using universal primers MBTuni-12 (5′-ACGCGTGATCAGCAAAAGCAGG) and MBTuni-13 (5′-ACGCGTGATCAGTAGAAACAAGG) as described previously [13]. Illumina MiSeq Technology was used for full-genome sequencing. The Nextera XT DNA Sample Preparation Kit (Illumina, San Diego, CA, USA) was used to generate multiplexed paired-end sequencing libraries, according to the manufacturer’s instructions. Each genome segment was assembled utilizing the comprehensive data analysis platform of Fusion Genomics Corporation (http://fusiongenomics.com, accessed on 7 May 2021) and the DNAstar SeqMan NGen software (Version 17; DNASTAR, Inc, Madison, WI, USA).

### 2.2. Nucleotide Sequences Used in the Study

Various sequence datasets were used throughout this study. For the origins of Mexican lineage H5N2 AIV gene segments, the sequences of initial Mexican H5N2 AIV strain A/chicken/Mexico/31381–7/1993 isolated in October of 1993 were used for BLAST with the output of 500 sequences. After removing identical sequences and sequences that did not contain full-length coding regions, the numbers of sequences when combined with recent Mexican lineage H5N2 AIV are as follows: PB2 (*n* = 334), PB1 (*n* = 335), PA (*n* = 324), HA (*n* = 384), NP (*n* = 324), NA (*n* = 305), M (*n* = 269), and NS (*n* = 294). All publicly available complete genome sequences and complete HA gene sequences of Mexican lineage H5N2 AIV were downloaded from the Influenza Research Database (IRD; https://www.fludb.org/), accessed on 29 November 2021 and GenBank (https://www.ncbi.nlm.nih.gov/genbank/), accessed on 29 November 2021. This leads to datasets of 189 complete full genome sequences and 284 complete H5 gene sequences that were used in this study.

### 2.3. Sequence Analyses

The nucleotides in the coding regions of segments 1 (PB2), 2 (PB1), 4 (HA), 5 (NP), and 6 (NA) were aligned using Muscle in MEGA-X [14]. The full nucleotide sequences of segments 3 (PA, and PA-X), 7 (M1 and M2), and 8 (NS1 and NS2) were also aligned using Muscle, and the sequences were edited such that all of the codons in the first open reading frame (ORF) were followed by the remaining codons in the second ORF in MEGA-X [14]. To infer phylogenetic trees, the maximum likelihood (ML) approach was applied by using RAxML v8.2.12 [15], with a GTR plus gamma substitution model and 1000 bootstrap iterations. Trees were rooted on the oldest sequence.

A Bayesian approach was implemented to explore additional evolutionary information. TempEst v1.5.3 was used to examine temporal signals in each dataset [16], taking an ML tree reconstructed by RAxML and the date of virus collection as input. The rate of nucleotide substitution per site per year (subs/site/year) and the time to the most recent common ancestor (tMRCA) of Mexican lineage H5N2 AIV strains were estimated for each gene segment using the Bayesian Markov Chain Monte Carlo (BMCMC) method in the program BEAST, version 2.6.6 [17]. Uncertainties in the estimates were summarized as the highest posterior density (HPD) intervals. The best-fit nucleotide substitution model was determined for each gene segment by MEGA-X software [14]. For all datasets, an uncorrelated log-normal relaxed molecular clock model was used, as it has been shown to best reflect the complex population dynamics of influenza A viruses [18]. The age of the viruses was defined as the date of sample collection. For the dataset, at least two independent BEAST analyses were run for 100 million generations and sampling every 10,000 generations. Convergences and effective sample sizes (ESS) of the estimates were checked using Tracer v1.7.2 (http://tree.bio.ed.ac.uk/software/tracer, accessed on 1 December 2021). All parameter estimates for each run showed ESS values >200. The nonparametric Coalescent Bayesian Skyline Plot (BSP) model [19] was used to measure the change in effective population size over time.

To estimate reassortment events, a coalescent-based phylogenetic network model was employed to allow the reassortant network and the embedding of each segment tree within the network to be jointly inferred with CoalRe [20].

### 2.4. Intravenous Pathogenicity Index of 2016–2019 Mexican H5N2 AIVs

All animal experiments were carried out according to the Mexican Regulations for Animal Welfare. Based on H5 HA cleavage site sequences, a total of 16 viruses with the amino acid motif of PQKRKR/G at the cleavage site were selected for the intravenous pathogenicity index (IVPI) test. To determine the IVPI of field isolates, 10 specific pathogen-free (SPF) chickens (4–6 weeks old) were inoculated intravenously with 0.1 mL of a 1:10 dilution of virus in sterile isotonic saline solution according to the OIE standard protocol [4]. Infected chickens were observed daily for clinical signs and mortality for 10 days. The severity of clinical signs was assessed using a standard pathogenicity index (PI) as recommended [4]. Briefly, healthy chickens were scored with 0. Chickens showing one clinical sign (e.g., ruffled feather, depression, nervous signs, diarrhea, edema, hemorrhages, or cyanosis in the unfeathered parts such as shanks, comb, or wattle) were given a score of 1, and chickens exhibiting at least two clinical signs were scored with 2. Dead chickens were given a score of 3 until the termination of the experiment. The PI was calculated using the sum of daily arithmetic means of all birds divided by ten (number of observation days) in each group. The virus is classified as a high pathogenic avian influenza virus if the IVPI value is greater than 1.2. In this study, all selected 2016–2019 Mexican H5N2 AIVs were classified into low pathogenic pathotypes.

### 2.5. Analysis of Selection Pressure

Site-specific selection pressures for all segments of the Mexican H5N2 AIVs were measured as nonsynonymous (dN)–synonymous (dS) nucleotide substitutions per site. The differences were estimated using the SLAC (Single-Likelihood Ancestor Counting) [21], MEME (Mixed Effects Model of Evolution) [22], and FUBAR (Fast, Unconstrained Bayesian AppRoximation) [23] methods available at the Datamonkey website [24]. A cut-off *p*-value to classify a site as positively or negatively selected was set at 0.1 for SLAC and 0.01 for the MEME method. The cut-off value for the posterior probability in the FUBAR method was set at 0.9 to reflect a positive or negative selection at a given site.

### 2.6. 3D Structural Analyses

Molecular graphics coordinates of the H5 HA crystal structure (PDB #2FK0) [25] from A/Vietnam/1203/04 were performed using the UCSF Chimera package from the Resource for Biocomputing, Visualization, and Informatics at the University of California, San Francisco [26]. The resulting images were imported into Adobe Photoshop for editing.

## 3. Results

### 3.1. Origins of the Mexican Lineage H5N2 AIV

**Figure 1:**
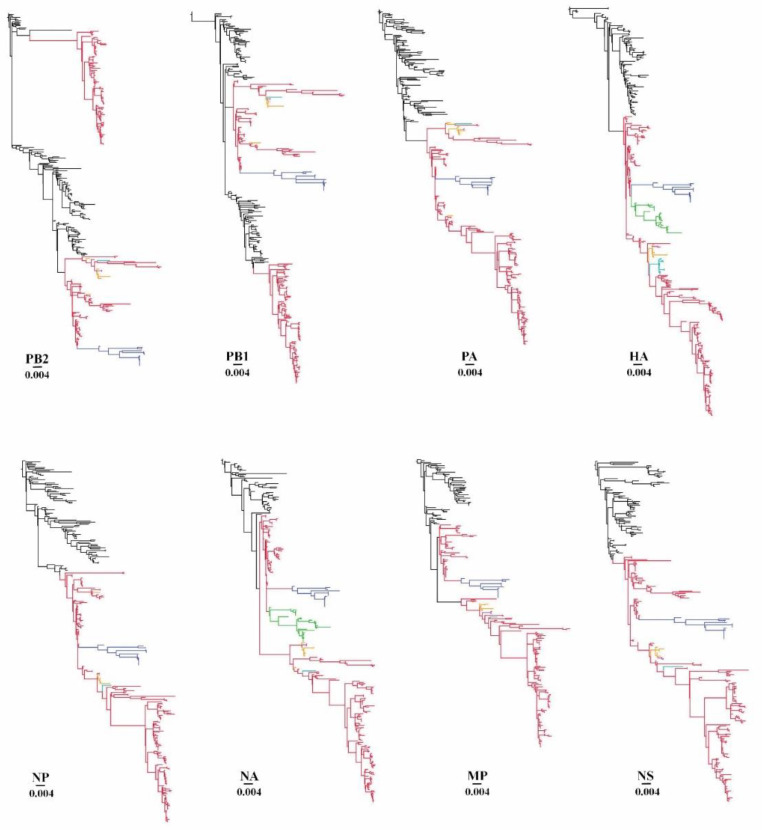
Evolutionary relationships of Mexican lineage H5N2 AIV sequences. The phylogenetic trees for each genome segment were inferred using RAxML. Black branches on the phylogenies represent various subtypes of AIV from the North American wild bird gene pool, while branches leading to enzootic viruses isolated from different geographic regions are color-coded as follows: red, Mexico; yellow, Guatemala; purple, EI Salvador; blue, Dominican Republic; green, Taiwan; cyan, Japan. Horizontal branch lengths are drawn to scale (nucleotide substitutions per site). Each segment tree is rooted on the closest related wild bird AIV sequence in the database.

The origins of each of the eight genomic segments of Mexican H5N2 AIV were determined from sequence datasets that comprised AIV sequences related to the initial Mexican H5N2 virus (A/chicken/Mexico/31381–7/1993) by BLAST and sequences generated in this study. As shown in Figure 1, each segment exhibited a monophyletic origin derived from the North American wild bird AIVs of various subtypes, with the exceptions of PB2 and PB1, which showed two separate introductions from the North American wild bird AIV gene pool viruses.

The ML phylogenetic trees showed that the Mexican H5N2 AIV had spread to the neighboring countries of Guatemala, EI Salvador, and the Dominican Republic, and had reached Japan and Taiwan in East Asia since its first isolation in October of 1993 [7]. Consistent with previous reports, the origins of all eight genome segments of Mexican lineage H5N2 were detected in poultry in Guatemala, EI Salvador, the Dominican Republic, and Japan but only HA and NA segments of Mexican lineage H5N2 in poultry of Taiwan [9,10,11,12].

### 3.2. The Evolutionary Rate of Mexican Lineage H5N2 AIV

Rates of nucleotide substitution and the times to the most recent common ancestor (tMRCAs) were estimated using a Bayesian coalescent approach for each genome segment separately. The means and 95% highest posterior density (95% HPD) intervals of all genomic segments are shown in Table 1. For the entire viral population, nucleotide substitution rates were high for the gene encoding the surface glycoproteins, with a mean rate of 5.69 × 10^−3^ sub/site/year (95% HPD, 5.32 × 10^−3^ to 6.06 × 10^−3^) for the HA gene and 5.49 × 10^−3^ sub/site/year (95% HPD, 5.09 × 10^−3^ to 5.91 × 10^−3^) for the NA gene. The estimated rates for the other genome segments ranged from 3.59 × 10^−3^ to 4.43 × 10^−3^ sub/site/year. The mean tMRCA estimations showed that Mexican lineage H5N2 AIV originated around 1990 (95% HPD between 1987 and 1993; Table 1), not long before the first isolation in 1993 in Mexico.

### 3.3. Protein-Level Evolution and Selection Analysis of Mexican Lineage H5N2 AIVs

Our analysis reveals that the HA, NA, M2, NS1, and NS2 genes have accumulated more nonsynonymous substitutions per site compared to the genes encoding polymerase complex (PB2, PB1, and PA), PA-X, and NP, which exhibited lower nonsynonymous substitutions per site (Supplementary Figure S1)

Various amino acid (aa) lengths of PB1-F2 were present within the dataset, including 90 aa (*n* = 49), 87 aa (*n* = 87), 79 aa (*n* = 2), and 52 aa (*n* = 27). Notably, PB1-F2 with 87 aa was only observed in 2016–2019 viruses. Out of 189 viruses, twenty-four viruses showed a non-functional PB1-F2 protein due to a mutation that resulted in an early stop codon in the PB1-F2 open reading frame.

**Figure 2:**
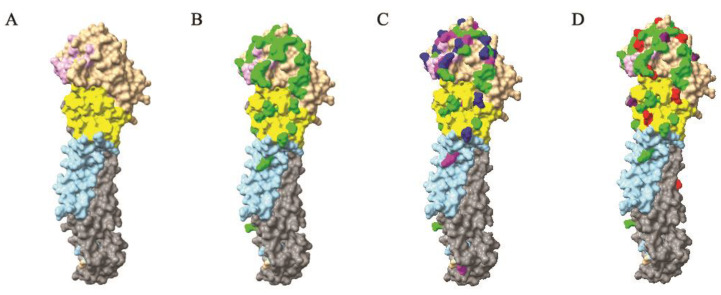
Antigenic variation, positive selection, and glycosylation in the 2016–2019 Mexican H5N2 AIVs mapped onto one H5 HA protomer. The H5 HA crystal structure is manipulated with Chimera and shown in the surface format using the structure from A/Vietnam/1203/04 (PDB #2FK0). (**A**) The globular head consists of the receptor-binding domain (RBD, tan, aa 110–262) and vestigial esterase domain (VED, yellow, aa 42–109, aa 263–272). The stalk domain contains regions of the N-terminus (aa 1–41) and the C-terminus of HA1 (sky blue, aa 273–330) and a membrane-proximal helix-rich region of HA2 (gray, aa 384–515). The receptor-binding site is shown in plum (HA1 129–133, 184–186, 217–224). (**B**) The known antigenic residues are shown in green. (**C**) The antigenic variations identified in the H5 HA protein of the 2016–2019 Mexican H5N2 AIVs against the HA protein of the two vaccines, A/chicken/Guanajuato/CPA-20966–15-VS/2015 (gene accession no. MH158232) and the recombinant NDV vector vaccine strain A/chicken/Mexico/435/2005 (gene accession no. FJ864690), are shown in magenta. Amino acid substitutions found in > 90% isolates are shown in blue. (**D**) Positively selected residues in the H5 HA protein of the 2016–2019 Mexican H5N2 AIVs are shown in red, and three glycosylated residues (aa 84, aa 126, and aa 162) found in later viruses but not early viruses are shown in purple. H5 numbering is used for amino acid position.

The selection pressure acting on the eight segment genes was assessed using the SLAC, FUBAR, and MEME methods (see Materials and Methods). The analysis revealed that the vast majority of codons were subject to purifying selection. Positive selections were detected in PB2 (1 residue), PB1 (3 residues), PA (2 residues), PA-X (4 residues), HA (12 residues), NP (1 residue), NA (4 residues), M2 (4 residues), NS1 (8 residues), and NS2 (2 residues) (Table 2). The biological functions of most of these positively selected residues are not known. However, six positively selected amino acids of H5 HA at positions 72, 123, 155, 181, 185, and 194 (H5 numbering, Figure 2D) are critical residues that are involved in antibody binding. Previous studies have shown that changes in amino acids at positions 123, 155, and 186 to proline, asparagine, and glycine, respectively, conferred increased binding to sialic acid-alpha2, 6-galactose (Siaα2, 6Gal) in genetic experiments of H5N1 viruses, and the mutant virus with the introduction of asparagine at position 155 showed a 100 fold reduction in the lethality of wild-type virus [27,28,29].

### 3.4. Estimate of Reassortment

**Figure 3:**
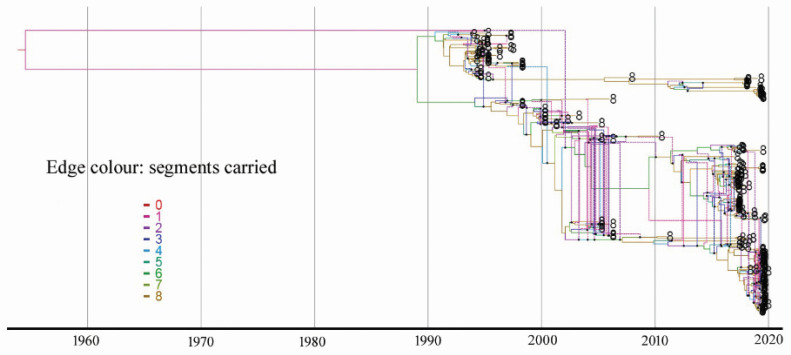
Estimate of reassortment network. Eight segment trees were embedded within the networks using CoalRe. Network edges are colored by segments carried.

Phylogenetic trees were constructed using full genome sequences of all Mexican lineage H5N2 AIVs available in public databases such as GenBank (*n* = 83). In addition, we generated 106 full genome sequences of Mexican lineage H5N2 AIVs collected between 2016 and 2019. Supplementary Figure S2 shows the maximum likelihood approach-based trees for each segment generated using RAxML. Trees were rooted with A/chicken/Mexico/31381–7/1993, as this was the oldest H5N2 virus in the dataset. To visualize incongruence, the phylogenetic position of each sequence (colored according to the origin of its HA) was traced across all seven trees. We did not observe incongruence within or between the Dominican Republic H5N2 clade and Guatemala/EI Salvador H5N2 clade in all eight of the segments, suggesting no reassortment had occurred in these two clades. Incongruence was also not observed between Mexican H5N2 viruses and the Dominican Republic H5N2 viruses. However, we observed clear incompatibilities among Mexican H5N2 AIV segments in all seven ML trees compared with HA phylogenies in 1994, 1995, 1998, 2005, 2006, 2016, 2017, and 2019, suggesting the occurrence of reassortment. This reassortment was further assessed using a coalescent-based approach to explicitly model the joint coalescent and reassortment process by embedding eight segment trees within the network (Figure 3). The inferred reassortment rate of the Mexican lineage H5N2 is 0.33 per lineage and year.

### 3.5. Diversity of the Mexican Lineage H5N2 AIV and HA Cleavage Sites

**Figure 4:**
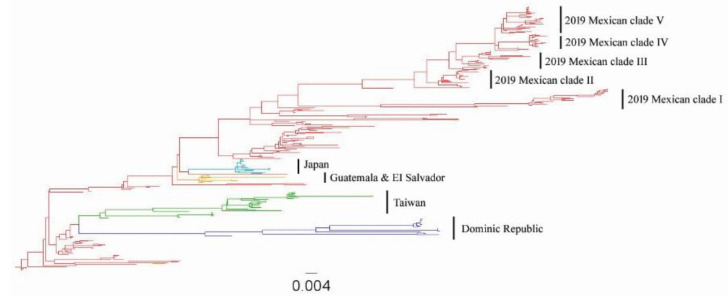
ML phylogenetic tree of the H5 HA genes of 284 Mexican lineage H5N2 AIVs collected between 1994 and 2019. Phylogenies are colored by the origin of the country. Red, Mexico; blue, Dominic Republic; green, Taiwan; yellow, Guatemala and EI Salvador; cyan, Japan. Distinct clades in 2019 Mexican H5N2 AIVs are denoted as clades I to V.

Previously, the H5 HA1 and fusion peptide sequences were used to study the evolution of Mexican lineage H5N2 AIV [6,7,30,31,32]. In this study, we conducted phylogenetic analysis by including all publicly available complete H5 HA sequences on a global scale (*n* = 284). Figure 4 shows that the Mexican H5N2 AIVs had formed at least five distinct clades in 2019. The four independently diversified clades from Japan, EI Salvador/Guatemala, Taiwan, and the Dominican Republic were nested within early Mexican sequences. We performed a regression of root-to-tip genetic distances on the phylogenetic tree for complete HA sequences against sampling dates (Supplementary Figure S3). This revealed a strongly linear accumulation of nucleotide divergence over time (R^2^ = 0.90). The HA sequence displayed a strong clock signal. The epidemic history of the Mexican lineage H5N2 AIV from 1994 to 2019 was analyzed using the Bayesian skyline plot (BSP) that depicts changes in the estimated effective population size through time. The BSP showed the enzootic circulation of the viruses in Mexico despite the vaccination program launched in 1995. The effective population size peaked in 2005 and was maintained at a high level until 2015 (Figure S4). Direct comparison between sequences shows that HA genetic diversity was higher in 2017–2019 and was five times that of viruses isolated in 1994 (Figure S5).

Examining the HA cleavage site has shown that multi-basic amino acids at the cleavage site which determines high pathogenicity in poultry had only been exhibited in the 1994 Puebla isolates (PQRKRKTR/G) and 1995 Queretaro isolates (PQRKRKTR/G and PRKRKRKTR/G) in Mexico and one 2014 isolate (PKREKREKR/G) in Taiwan. The basic amino acids at the cleavage site were counted including the Arg residue (−1 position) immediately preceding the junction between HA1 and HA2 subunits but excluding the −4 position found in the LPAI cleavage site consensus motif. Within the examined dataset, a cleavage site containing one basic amino acid was observed in the early isolates of Mexico (1994–2007), Japan (2005–2006), EI Salvador/Guatemala (2000–2003), and the Dominican Republic (2007). A cleavage site containing two to three basic amino acids was observed in recent isolates of Mexico (2016–2019) and the Dominican Republic (2017–2019), while it showed in all isolates of Taiwan (2003–2019).

### 3.6. Antigenic Evolution of H5N2 AIVs in Mexico

Since the first isolation of a low pathogenic H5N2 virus in chicken in 1993 in Mexico, H5N2 AIV has become enzootic in poultry despite an extensive vaccination program launched in 1995. To investigate the antigenic evolution of Mexican H5N2 AIVs, the full-genome sequences of 106 Mexican H5N2 AIVs were generated. These viruses are distributed throughout 15 states in Mexico and detected by the AIV surveillance system between 2016 and 2019 (Supplementary Table S1). Phylogenetically, the Mexican H5N2 HA gene had diversified from the initial two lineages (i.e., Puebla lineage and Jalisco lineage) between October 1993 and January 1995 to at least five distinct clades in 2019 (Figure 4).

**Figure 5:**
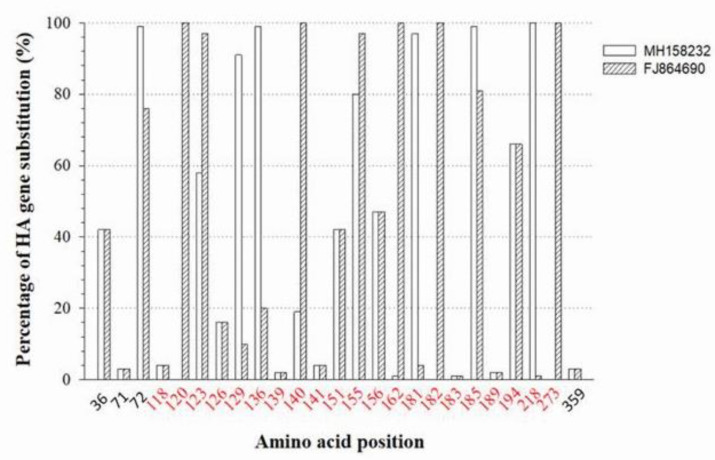
Amino acid substitutions at the known antigenic sites of the H5 HA protein. The degree of amino acid substitutions is summarized from 106 H5 HA genes of Mexican H5N2 AIVs collected between 2016 and 2019 against H5 HA proteins of the vaccine strain A/chicken/Guanajuato/CPA-20966-15-VS/2015 (gene accession no. MH158232) and the recombinant NDV vector vaccine strain A/chicken/Mexico/435/2005 (gene accession no. FJ864690). The residues located within the receptor-binding domain are colored in red.

Forty-five critical residues in the H5 HA protein have been determined by monoclonal antibody escape mutant studies [33,34,35,36,37,38,39]. Among these, 42 residues are located in the HA1 subunit and three residues reside in the HA2 subunit (Figure 2B). The H5 influenza viral HA protein is structurally divided into two portions [40]: (1) globular head, which contains the receptor-binding domain (RBD) and the vestigial esterase domain (VED); (2) stalk, which contains an N-terminus and a C-terminus of the HA1 subunit and a membrane-proximal helix-rich stem structure of the HA2 subunit (Figure 2A). When comparing the HA genes of the currently used inactivated vaccine strain, A/chicken/Guanajuato/CPA-20966–15-VS/2015 (H5N2) and the recombinant NDV vector with H5 insert of A/chicken/Mexico/435/2005, the substitution of antigenic residues in the 2016–2019 Mexican H5N2 HA gene mainly occurred in the receptor-binding domain (80%) compared with HA of the recombinant vector vaccine and 85% compared with inactivated vaccine, followed by the vestigial esterase domain (7% and 10%, respectively), and the stalk (13% and 5%, respectively). Out of the reported 45 critical residues, twenty-five residues were substituted in 2016–2019 Mexican H5N2 viruses (Figure 5). The substitution rates are higher at the residues of the receptor-binding domain with exception of residues 71, 118, 139, 141, 183, 189, and 359. The structural locations of these substituted residues are depicted in Figure 2C.

We predicted potential N-glycosylation sites of the H5 HA protein by using NetNGlyc 1.0 server (http://www.cbs.dtu.dk/services/NetNGlyc, accessed on 2 January 2022). Analysis of 210 complete Mexican H5 HA sequences showed 18 potential N-glycosylated sites among HA proteins of 1994 to 2019 isolates (Table 3). Residues that are glycosylated at positions 10, 23, 236, 480, and 539 (H5 numbering) are dominant in the entire dataset. Notably, sites glycosylated at positions 84 (112/210, 53.3%), 126 (153/210, 72.9%), and 163 (172/210, 81.9%) are located at or near antigenic sites (Figure 2D). Glycosylation at these three sites is not found in HA proteins of early isolates but is found in the 2005–2019 isolates. Glycosylation at site 84 is absent in the HA of the above two vaccines.

## 4. Discussion

Since the discovery of H5N2 AIV in Mexico in 1993–1994, most studies have focused on the genetic characterization of the HA gene and the pathogenic characterization of infected chickens [6,7,30,31,32,41]. In this study, we generated 106 complete genome sequences of Mexican H5N2 AIVs and compiled them with publicly available complete Mexican lineage H5N2 genome sequences to carry out comprehensive genetic and phylodynamic analyses to elucidate its evolutionary history. In particular, we analyzed the H5 HA gene for its antigenic evolution within the overall history of Mexican H5N2 AIVs.

Our results confirm previous findings and provide new insight into the origins, epidemiology, and evolution of Mexican lineage H5N2 AIVs. We determined with greater accuracy than previous reports the origins of Mexican lineage H5N2 AIVs. All the gene segments of the Mexican lineage H5N2 AIV have originated from the North American wild bird gene pool viruses with a monophyletic introduction except for PB2 and PB1, whose gene segments originated from two separate introductions. The phylogenies for each of the segments suggest that the genetic constellation arose through multiple reassortment events. Although the exact source of the initial Mexican H5N2 LPAI outbreak has never been identified, all of the isolates showed 20 amino acid deletions in the stalk of the NA protein compared to the N2 NA protein of aquatic birds along with eight additional glycosylation sites in the HA globular head. These molecular signatures have frequently appeared during the adaptation of viruses from aquatic avian hosts to domestic poultry [42,43]. The mean tMRCA of each genome segment was estimated to be around 1990, suggesting that the period in which H5N2 viruses originating from aquatic birds had undergone significant selective pressure in the chicken population, leading to definite changes in both the HA and the NA proteins during the adaptation process.

Our analysis also reveals important epidemiological features of the Mexican lineage H5N2 AIVs. The global epidemic history of Mexican lineage H5N2 AIVs from 1994 to 2019 showed an increase in the effective numbers of infections in 2005 and maintained at a high level until 2015. The repercussions of that increase are attributed to the spreading of the virus to neighboring countries and East Asia via unclear pathways. The virus was first detected in 2003 in Taiwan with HA and NA genes derived from the Mexican lineage H5N2 virus and internal genes from the local enzootic H6N1 virus [10]. Subsequently, this virus was detected in Japan in 2005 [11] and the Dominican Republic in 2007 [12], with the virus tending to establish local lineages that evolve in situ. We were unable to define the transmission of the circulating virus from poultry to wild birds due to the lack of full genome sequence data of Mexican wild bird populations. Generating full genome sequence data from these wild bird populations would assist in understanding the spreading of Mexican lineage H5N2 AIVs to other geographic regions.

The evolution of Mexican lineage H5N2 AIVs has involved genetic substitutions and reassortments. Our data show higher nucleotide substitution rates and elevated mean dN/dS values for HA and NA. This may have resulted from immune pressure caused by a previous infection or as a result of vaccination. These rates are twofold lower than those reported for Mexican H5N2 viruses isolated in 1994–2002 [30]. High evolutionary rates have been reported during the establishment of new virus lineages (for example, see [44,45,46]), which may reflect the preferential selection of mutants that provide an advantage within a new host. Elevated mean dN/dS values for M2, NS1, and NS2 are consistent with positive selection for host-adaptive mutations. Further experimental work is needed to clarify any functional nuances of the coding changes. Although the biological function of PB1-F2 in the avian host is largely unknown, truncated PB1-F2 is noticeable when the AIV has primarily circulated in poultry populations [47,48]. The PB1-F2 polymorphisms in the Mexican lineage H5N2 AIVs require further experimental studies to understand their evolutionary benefits if any. Previous studies have shown that intrasubtypic reassortment is not an uncommon event among AIVs [49,50,51]. We have observed reassortment events involving all segments of the Mexican H5N2 AIVs, which are most likely to be intrasubtypic reassortment. However, the fact that there is co-circulation of H7N3 HPAIV [52] and H5N2 LPAIV in Mexican poultry would require further assessment for heterosubtypic reassortment between these two viruses. Nevertheless, Youk et al. have studied the evolution of Mexican H7N3 HPAIV and found no evidence of reassortment events detected in 2015–2017 H7N3 HPAIV with the circulating H5N2 LPAIV within Mexican poultry [4,53].

Our data showed that Mexican lineage H5N2 AIVs are enzootic in Mexico, the Dominican Republic, and Taiwan. This H5N2 virus has undergone rapid genetic diversification. It is five times more diversified in recent isolates than the initial isolates in 1994. Notably, Mexican H5N2 AIVs have diversified into at least five clades as of 2019. Analysis of the H5 HA gene showed a low pathogenic sequence motif at the cleavage site for Mexican lineage H5N2 AIVs except for some early 1994–1995 isolates in Mexico and one 2014 isolate in Taiwan. The H5 HA cleavage site sequence of 2016–2019 Mexican H5N2 viruses (*n* = 106) shows typical LP polymorphism of two basic amino acids: PQRKTR/G (*n* = 87), PQRETR/G (*n* = 1), and PQKGKR/G (*n* = 1), and three basic amino acids: PQKRKR/G (*n* = 16) and SQKRKR/G (*n* = 1). However, examination of viral pathogenicity by intravenous pathogenicity indexing (IVPI) in four- to eight-week-old chickens of selected Mexican H5N2 isolates with cleavage site sequence of PQKRKR/G demonstrated low pathogenicity of the virus (Supplementary Table S1). Since H5N2 HPAIV was officially eradicated in Mexico in mid-1995, no H5N2 HPAIV has been detected as of 2019. The conversion of LPAIV to HPAIV occurs via changes at the HA cleavage site motif by either the substitution of non-basic amino acids, which is likely through polymerase duplication or slippage events during RNA replication, or the recombination with the insertion of cellular or viral amino acids [54,55,56,57,58,59]. However, these changes are random events, and when and if this will occur remains unpredictable. Similar scenarios have been observed, such as in ostriches infected with H7Nx LPAIV, where the conversion of LP H7 virus to HP H7 virus was never previously reported, despite frequent detection during extended periods of circulation [60]. In addition, vaccination against H5N2 LPAIV is in effect vaccinating against HPAIV. An inactivated H5N2 vaccine was used in Mexico as a result of the widespread HPAIV outbreaks caused by the H5N2 virus that began in December 1994. A total of 383 million doses of inactivated oil-emulsified vaccine, made from a 1994 precursor H5N2 LPAI virus, were used to control the spreading of H5N2 HPAIV [61]. The ability of the virus to spread easily must, to some extent, be related to the amount of virus released by the respiratory or intestinal route. The highly pathogenic viruses cause extremely rapid deaths in these birds, and the relatively little virus may be excreted due to vaccination.

The implementation of a vaccination program in 1995 within Mexican poultry coupled with improved biosecurity measures and active surveillance has eliminated the occurrence of high pathogenic H5N2 viruses. However, the low pathogenic H5N2 viruses continue to persist in Mexican poultry, as demonstrated in this study. The HA protein is the major antigenic component of the virus, and currently, forty-five amino acids are characterized as critical residues for antibody–antigen binding by escape mutant studies [33,34,35,36,37,38,39]. In this study, the genetic drift acting on putative antigenic sites of the HA protein of 2016–2019 Mexican H5N2 AIVs were assessed by comparing them with the HA proteins of the currently used inactivated vaccine seed strain (A/chicken/Guanajuato/CPA-20966–15-VS/2015) and a recombinant NDV vector strain (A/chicken/Mexico/435/2005). The analyses showed extensive amino acid substitutions at the antigenic sites. These changes occurred predominantly in the receptor-binding domain (> 80% of observed amino acid changes), which contains major antigenic sites. Protection against influenza viruses depends mainly on vaccination and naturally acquired immunity, but the rapid antigenic evolution of these viruses allows them to escape population immunity. Glycosylation is another strategy utilized by influenza viruses to escape recognition by the immune system [62,63]. Three glycosylated residues at positions 84, 126, and 163 that are located at or near antigenic sites were not observed in the early isolates but were observed in the later viral isolates. This may also contribute to antigenic evolution by reducing the ability of the host to mount an effective immune response to these more recent H5N2 viruses. Since the inactivated vaccine was updated in 2015, the effective infected population size dropped. However, we have observed the starting of a marked increase in 2019. The H5N2 virus drift variants may have resulted from the positive selection of spontaneous mutants by neutralizing antibodies. These variants can therefore no longer be neutralized by antibodies to the parental strains. In addition, vaccine coverage might have also influenced the success of the eradication of H5N2 LPAIV in Mexican poultry. In general, vaccination programs should aim to have 70 to 80% coverage with adequate levels of protective antibody titers. However, Swayne and colleagues reported that the average H5 vaccine coverage rate in Mexican poultry from 2002 to 2010 was around 28.8% [64]. Swayne et al. also examined the efficacy of different inactivated H5 vaccines made up of different H5 subtype viruses obtained from four continents against challenge with HPAI H5N2 isolate from Mexico [65]. In their studies, vaccination was able to protect against the clinical manifestation of the disease and also reduced virus shedding in oropharyngeal and cloacal swabs; however, the most consistent reduction in shedding was observed in groups where the vaccine and challenge viruses were similar.

In summary, we have used complete genome sequence data to perform a comprehensive analysis of the evolution of the Mexican lineage H5N2 AIVs since its emergence in 1993/1994. The evolutionary properties of the major antigenic determinants of the HA gene were analyzed against the two currently used vaccines in 2016–2019 Mexican H5N2 AIVs. Findings from this study suggest the need for vaccine updates and emphasize the importance of continuing molecular characterization of the antigenic determinant for its antigenic and phenotypic changes.

## Figures and Tables

**Table 1:** Estimates of nucleotide substitution rates and tMRCA for each gene segment of Mexican lineage H5N2 AIVs.

Segment	Substitution Rate (Subs/Site/Year)		tMRCA (Year)	
	Mean (×10^−3^)	95% HPD (×10^−3^)	Mean	95% HPD (×10^−3^)
PB2	4.32	4.01–4.64	1990.80	1988.88–1992.34
PB1	3.79	3.56–4.04	1991.15	1989.03–1992.62
PA	3.80	3.52–4.06	1990.75	1988.30–1992.66
HA	5.69	5.32–6.06	1990.27	1987.92–1992.21
NP	3.78	3.50–4.08	1990.38	1987.08–1992.54
NA	5.49	5.09–5.91	1991.35	1988.04–1993.22
MP	3.59	3.27–3.89	1990.33	1987.43–1992.69
NS	4.43	4.10–4.76	1990.97	1988.83–1992.73

**Table 2:** Amino acid sites under putative positive selection detected using different analytical models.

Gene	Codon Position	SLAC		FUBAR		MEME	
		dN-dS	*p*-Value	dN-dS	Posterior Probability	*p*-Value	Number of Branches under Episodic Selection
PB2	191			1.68	0.90		
PB1	296					0.00	1
	746	2.49	0.09	1.59	0.91		
	757					0.00	1
PA	210	3.56	0.08				
	489	5.02	0.05	5.91	0.98	0.00	12
PA-X	193	3.48	0.08	2.11	0.98		
	216	3.32	0.06	1.91	0.97		
	218	2.85	0.09	1.57	0.95		
	221	2.74	0.09				
H5	88 (72) *	2.44	0.06	2.06	0.97		
	139 (123)	3.25	0.01	2.19	0.99	0.00	9
	153 (137)	5.09	0.00	6.33	1.00	0.00	5
	171 (155)	4.41	0.00	3.59	0.99	0.00	7
	174 (158)					0.00	11
	194 (178)	2.54	0.07	2.00	0.95		
	197 (181)	2.96	0.04	1.94	0.95		
	201 (185)					0.00	2
	202 (186)					0.00	4
	210 (194)	4.96	0.01	5.20	0.97	0.00	5
	286 (270)	2.16	0.08	1.44	0.92		
	392 (376)	2.43	0.06	1.91	0.96		
NP	452	3.44	0.07	2.47	0.95		
N2	43	2.61	0.08	1.75	0.97		
	58	3.55	0.06				
	362					0.00	1
	449					0.00	3
M2	13			2.49	0.98		
	20			1.63	0.94		
	28			4.81	0.95		
	89			1.83	0.94		
NS1	63	3.04	0.06	2.57	0.97		
	64			1.72	0.92		
	176			2.20	0.93		
	205			1.81	0.94		
	210	2.60	0.08	2.11	0.96		
	215	4.24	0.02	4.94	0.98	0.01	6
	221			2.68	0.95		
	226			2.17	0.94		
NS2	14			3.58	0.96		
	44			2.74	0.92		

() *, H5 numbering.

**Table 3:** Potential N-glycosylation sites identified in HA protein of 210 Mexican H5N2 AIVs.

Amino Acid Position ^a^	Structural Location ^b^	Sequons ^c^	Percentage of Isolates (%)
10	stalk	N*N*ST	99.5
23	stalk	*N*VTV	99.5
45	VED	*N*GTK	1.9
54	VED	*N*CSV	2.4
72	VED	*N*VSE	1.4
84	VED	*N*PSN	53.3
126	RBD	*N*ASA	5.7
		*N*ASS	67.1
138	RBD	*N*GSS	0.5
161	RBD	*N*RTY	0.5
163	RBD	*N*YTN	81.9
165	RBD	*N*NTN	14.3
193	RBD	*N*PST	1.0
		*N*PTT	0.5
236	RBD	*N*DSI	99.5
275	stalk	*N*ATC	1.4
286	stalk	*N*SSL	79.5
		*N*SSM	14.3
		*N*TSM	2.9
319	stalk	*N*VSQ	0.5
480 (484)	stalk	*N*GTY	100.0
539 (543)	CD	*N*GSL	100.0

^a^, H5 numbering. HPAI indicated in brackets; ^b^, VED (vestigial esterase domain), RBD (receptor binding domain), CD (cytoplasmic domain); ^c^, N-glycosylated site is in italic font with an underline.

## Data Availability

One hundred and six whole-genome sequences were obtained from active surveillance in Mexican poultry, which have GenBank accession numbers MZ564905-mz565408.

## Supplementary Materials

The following supporting information can be downloaded at: https://www.mdpi.com/article/10.3390/v14050958/s1, Table S1: 2016–2019 Mexican H5N2 AIVs used in this study. Figure S1: Mean dN/dS values for major coding regions of Mexican lineage H5N2 AIVs. Only full genomes of H5N2 AIVs were analyzed; Figure S2. Phylogenetic incongruence analysis. ML trees for each genomic segment were inferred from 189 full-genome H5N2 viruses using RAxML. The phylogenies of segments are colored according to the origin of their HA. The phylogenetic position of each sequence was traced across all seven trees; Figure S3. Clock-like nature of Mexican lineage H5N2 AIV evolution. Regression of root-to-tip genetic divergence against sampling date was performed based on a phylogeny of 284 complete HA sequences. Sequences are colored according to their geographic locations. Red, Mexico; blue, Dominic Republic; green, Taiwan; yellow, Guatemala and EI Salvador; cyan, Japan; Figure S4. Bayesian skyline plot showing the demographic history of Mexican lineage H5N2 AIV circulating globally. The H5 HA sequences from 284 H5N2 viruses were included in the analysis. The thick colored line is the mean estimate, and the 95% HPD is indicated by the shaded area; Figure S5. Temporal changes in genetic diversities of Mexican lineage H5N2 AIVs. The HA genes from 284 H5N2 viruses were used to calculate the genetic diversity (π) detected in each year.
