# Supplementary material for: Evolutionary Dynamics of Mexican Lineage H5N2 Avian Influenza Viruses
Source: Viruses. 2022 May 3;14(5):958. doi: 10.3390/v14050958 (PMC9146523; doi:10.3390/v14050958)
Supplement: Supplementary file 1 [file viruses-14-00958-s001.zip › Figure S1.pdf]

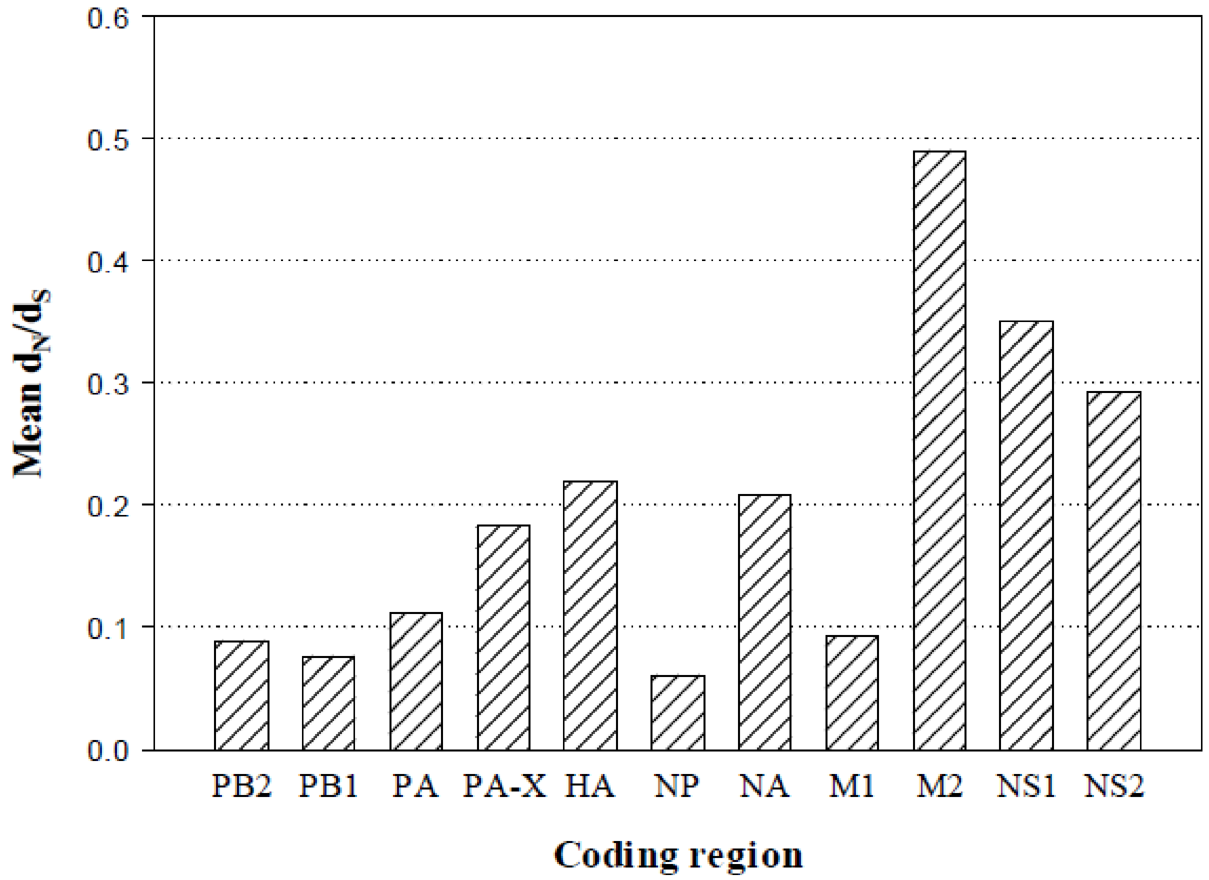

Figure S1. Mean dN/dS values for major coding regions of Mexican lineage H5N2 AIVs. Only full genomes of H5N2 AIVs were analyzed.
