# Supplementary material for: Evolutionary Dynamics of Mexican Lineage H5N2 Avian Influenza Viruses
Source: Viruses. 2022 May 3;14(5):958. doi: 10.3390/v14050958 (PMC9146523; doi:10.3390/v14050958)
Supplement: Supplementary file 1 [file viruses-14-00958-s001.zip › Figure S2.pdf]

**Figure S2.** Phylogenetic incongruence analysis. ML trees for each genomic segment were inferred from 189 full-genome H5N2 viruses using RAxML. The phylogenies of segments are colored according to the origin of their HA. The phylogenetic position of each sequence was traced across all seven trees.

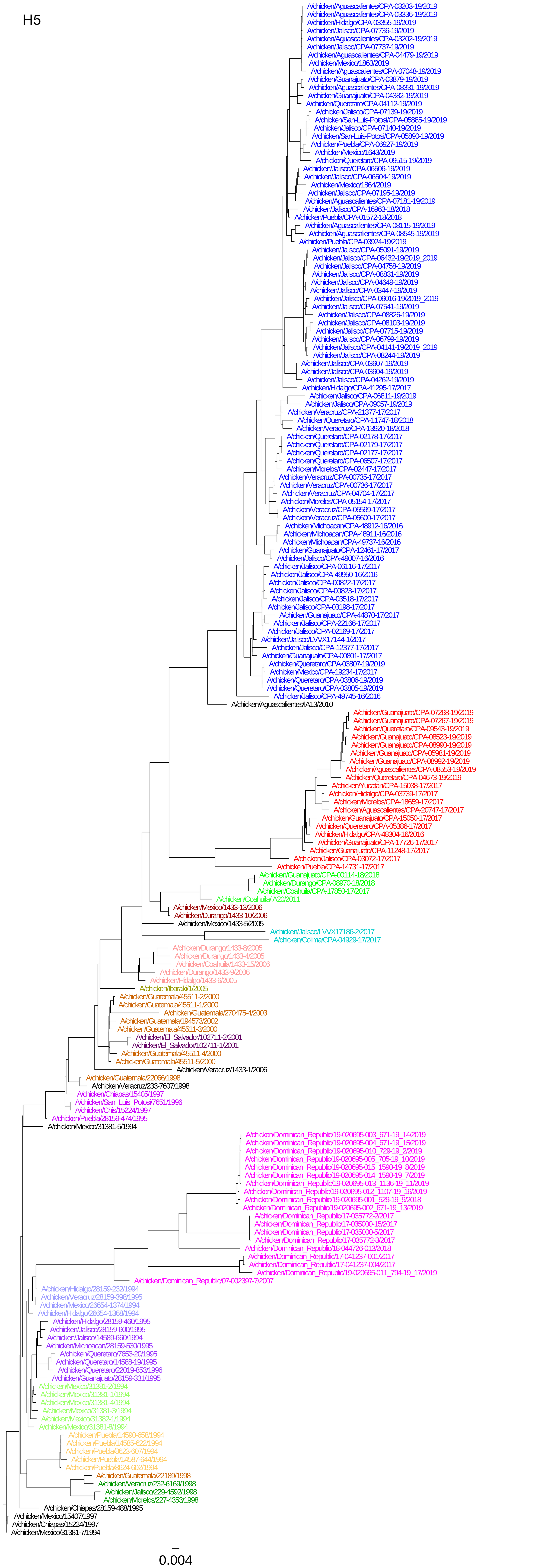
H5
A/chicken/Aguascalientes/CPA-03203-19/2019
A/chicken/Aguascalientes/CPA-03336-19/2019
A/chicken/Hidalgo/CPA-03355-19/2019
A/chicken/Jalisco/CPA-07736-19/2019
A/chicken/Aguascalientes/CPA-03202-19/2019
A/chicken/Jalisco/CPA-07737-19/2019
A/chicken/Aguascalientes/CPA-04479-19/2019
A/chicken/Mexico/1863/2019
A/chicken/Aguascalientes/CPA-07048-19/2019
A/chicken/Guanajuato/CPA-03879-19/2019
A/chicken/Aguascalientes/CPA-08331-19/2019
A/chicken/Guanajuato/CPA-04382-19/2019
A/chicken/Queretaro/CPA-04112-19/2019
A/chicken/Jalisco/CPA-07139-19/2019
A/chicken/San-Luis-Potosi/CPA-05885-19/2019
A/chicken/Jalisco/CPA-07140-19/2019
A/chicken/San-Luis-Potosi/CPA-05890-19/2019
A/chicken/Puebla/CPA-06927-19/2019
A/chicken/Mexico/1643/2019
A/chicken/Queretaro/CPA-09515-19/2019
A/chicken/Jalisco/CPA-06506-19/2019
A/chicken/Jalisco/CPA-06504-19/2019
A/chicken/Mexico/1864/2019
A/chicken/Jalisco/CPA-07195-19/2019
A/chicken/Aguascalientes/CPA-07181-19/2019
A/chicken/Jalisco/CPA-16963-18/2018
A/chicken/Puebla/CPA-01572-18/2018
A/chicken/Aguascalientes/CPA-08115-19/2019
A/chicken/Aguascalientes/CPA-08545-19/2019
A/chicken/Puebla/CPA-03924-19/2019
A/chicken/Jalisco/CPA-05091-19/2019
A/chicken/Jalisco/CPA-06432-19/2019_2019
A/chicken/Jalisco/CPA-04758-19/2019
A/chicken/Jalisco/CPA-08831-19/2019
A/chicken/Jalisco/CPA-04649-19/2019
A/chicken/Jalisco/CPA-03447-19/2019
A/chicken/Jalisco/CPA-06016-19/2019_2019
A/chicken/Jalisco/CPA-07541-19/2019
A/chicken/Jalisco/CPA-08826-19/2019
A/chicken/Jalisco/CPA-08103-19/2019
A/chicken/Jalisco/CPA-07715-19/2019
A/chicken/Jalisco/CPA-06799-19/2019
A/chicken/Jalisco/CPA-04141-19/2019_2019
A/chicken/Jalisco/CPA-08244-19/2019
A/chicken/Jalisco/CPA-03607-19/2019
A/chicken/Jalisco/CPA-03604-19/2019
A/chicken/Jalisco/CPA-04262-19/2019
A/chicken/Hidalgo/CPA-41295-17/2017
A/chicken/Jalisco/CPA-06811-19/2019
A/chicken/Jalisco/CPA-09057-19/2019
A/chicken/Veracruz/CPA-21377-17/2017
A/chicken/Queretaro/CPA-11747-18/2018
A/chicken/Veracruz/CPA-13920-18/2018
A/chicken/Queretaro/CPA-02178-17/2017
A/chicken/Queretaro/CPA-02179-17/2017
A/chicken/Queretaro/CPA-02177-17/2017
A/chicken/Queretaro/CPA-06507-17/2017
A/chicken/Morelos/CPA-02447-17/2017
A/chicken/Veracruz/CPA-00735-17/2017
A/chicken/Veracruz/CPA-00736-17/2017
A/chicken/Veracruz/CPA-04704-17/2017
A/chicken/Morelos/CPA-05154-17/2017
A/chicken/Veracruz/CPA-05599-17/2017
A/chicken/Veracruz/CPA-05600-17/2017
A/chicken/Michoacan/CPA-48912-16/2016
A/chicken/Michoacan/CPA-48911-16/2016
A/chicken/Michoacan/CPA-49737-16/2016
A/chicken/Guanajuato/CPA-12461-17/2017
A/chicken/Jalisco/CPA-49007-16/2016
A/chicken/Jalisco/CPA-06116-17/2017
A/chicken/Jalisco/CPA-49950-16/2016
A/chicken/Jalisco/CPA-00822-17/2017
A/chicken/Jalisco/CPA-00823-17/2017
A/chicken/Jalisco/CPA-03518-17/2017
A/chicken/Jalisco/CPA-03198-17/2017
A/chicken/Guanajuato/CPA-44870-17/2017
A/chicken/Jalisco/CPA-22166-17/2017
A/chicken/Jalisco/CPA-02169-17/2017
A/chicken/Jalisco/LVX17144-1/2017
A/chicken/Jalisco/CPA-12377-17/2017
A/chicken/Guanajuato/CPA-00801-17/2017
A/chicken/Queretaro/CPA-03807-19/2019
A/chicken/Mexico/CPA-19234-17/2017
A/chicken/Queretaro/CPA-03806-19/2019
A/chicken/Queretaro/CPA-03805-19/2019
A/chicken/Jalisco/CPA-49745-16/2016
A/chicken/Aguascalientes/IA13/2010
A/chicken/Guanajuato/CPA-07268-19/2019
A/chicken/Guanajuato/CPA-07267-19/2019
A/chicken/Queretaro/CPA-09543-19/2019
A/chicken/Guanajuato/CPA-08523-19/2019
A/chicken/Guanajuato/CPA-08990-19/2019
A/chicken/Guanajuato/CPA-05981-19/2019
A/chicken/Guanajuato/CPA-08992-19/2019
A/chicken/Aguascalientes/CPA-08553-19/2019
A/chicken/Queretaro/CPA-04673-19/2019
A/chicken/Yucatan/CPA-15038-17/2017
A/chicken/Hidalgo/CPA-03739-17/2017
A/chicken/Morelos/CPA-18659-17/2017
A/chicken/Aguascalientes/CPA-20747-17/2017
A/chicken/Guanajuato/CPA-15050-17/2017
A/chicken/Queretaro/CPA-05386-17/2017
A/chicken/Hidalgo/CPA-48304-16/2016
A/chicken/Guanajuato/CPA-17726-17/2017
A/chicken/Guanajuato/CPA-11248-17/2017
A/chicken/Jalisco/CPA-03072-17/2017
A/chicken/Puebla/CPA-14731-17/2017
A/chicken/Guanajuato/CPA-00114-18/2018
A/chicken/Durango/CPA-08970-18/2018
A/chicken/Coahuila/CPA-17850-17/2017
A/chicken/Coahuila/IA20/2011
A/chicken/Mexico/1433-13/2006
A/chicken/Durango/1433-10/2006
A/chicken/Mexico/1433-5/2005
A/chicken/Jalisco/LVX17186-2/2017
A/chicken/Colima/CPA-04929-17/2017
A/chicken/Durango/1433-8/2005
A/chicken/Durango/1433-4/2005
A/chicken/Coahuila/1433-15/2006
A/chicken/Durango/1433-9/2006
A/chicken/Hidalgo/1433-6/2005
A/chicken/Ibaraki/1/2005
A/chicken/Guatemala/45511-2/2000
A/chicken/Guatemala/45511-1/2000
A/chicken/Guatemala/270475-4/2003
A/chicken/Guatemala/194573/2002
A/chicken/Guatemala/45511-3/2000
A/chicken/El_Salvador/102711-2/2001
A/chicken/El_Salvador/102711-1/2001
A/chicken/Guatemala/45511-4/2000
A/chicken/Guatemala/45511-5/2000
A/chicken/Veracruz/1433-1/2006
A/chicken/Guatemala/22066/1998
A/chicken/Veracruz/233-7607/1998
A/chicken/Chiapas/15405/1997
A/chicken/San_Luis_Potosi/7651/1996
A/chicken/Chis/15224/1997
A/chicken/Puebla/28159-474/1995
A/chicken/Mexico/31381-5/1994
A/chicken/Dominican_Republic/19-020695-003_671-19_14/2019
A/chicken/Dominican_Republic/19-020695-004_671-19_15/2019
A/chicken/Dominican_Republic/19-020695-010_729-19_2/2019
A/chicken/Dominican_Republic/19-020695-005_705-19_10/2019
A/chicken/Dominican_Republic/19-020695-015_1590-19_8/2019
A/chicken/Dominican_Republic/19-020695-014_1590-19_7/2019
A/chicken/Dominican_Republic/19-020695-013_1136-19_11/2019
A/chicken/Dominican_Republic/19-020695-012_1107-19_16/2019
A/chicken/Dominican_Republic/19-020695-001_529-19_9/2018
A/chicken/Dominican_Republic/19-020695-002_671-19_13/2019
A/chicken/Dominican_Republic/17-035772-2/2017
A/chicken/Dominican_Republic/17-035000-15/2017
A/chicken/Dominican_Republic/17-035000-5/2017
A/chicken/Dominican_Republic/17-035772-3/2017
A/chicken/Dominican_Republic/18-044726-013/2018
A/chicken/Dominican_Republic/17-041237-001/2017
A/chicken/Dominican_Republic/17-041237-004/2017
A/chicken/Dominican_Republic/19-020695-011_794-19_17/2019
A/chicken/Dominican_Republic/07-002397-7/2007
A/chicken/Hidalgo/28159-232/1994
A/chicken/Veracruz/28159-398/1995
A/chicken/Mexico/26654-1374/1994
A/chicken/Hidalgo/26654-1368/1994
A/chicken/Hidalgo/28159-460/1995
A/chicken/Jalisco/28159-600/1995
A/chicken/Jalisco/14589-660/1994
A/chicken/Michoacan/28159-530/1995
A/chicken/Queretaro/7653-20/1995
A/chicken/Queretaro/14588-19/1995
A/chicken/Queretaro/22019-853/1996
A/chicken/Guanajuato/28159-331/1995
A/chicken/Mexico/31381-2/1994
A/chicken/Mexico/31381-1/1994
A/chicken/Mexico/31381-4/1994
A/chicken/Mexico/31381-3/1994
A/chicken/Mexico/31382-1/1994
A/chicken/Mexico/31381-8/1994
A/chicken/Puebla/14590-658/1994
A/chicken/Puebla/14585-622/1994
A/chicken/Puebla/8623-607/1994
A/chicken/Puebla/14587-644/1994
A/chicken/Puebla/8624-602/1994
A/chicken/Guatemala/22189/1998
A/chicken/Veracruz/232-6169/1998
A/chicken/Jalisco/229-4592/1998
A/chicken/Morelos/227-4353/1998
A/chicken/Chiapas/28159-488/1995
A/chicken/Mexico/15407/1997
A/chicken/Chiapas/15224/1997
A/chicken/Mexico/31381-7/1994
0.004

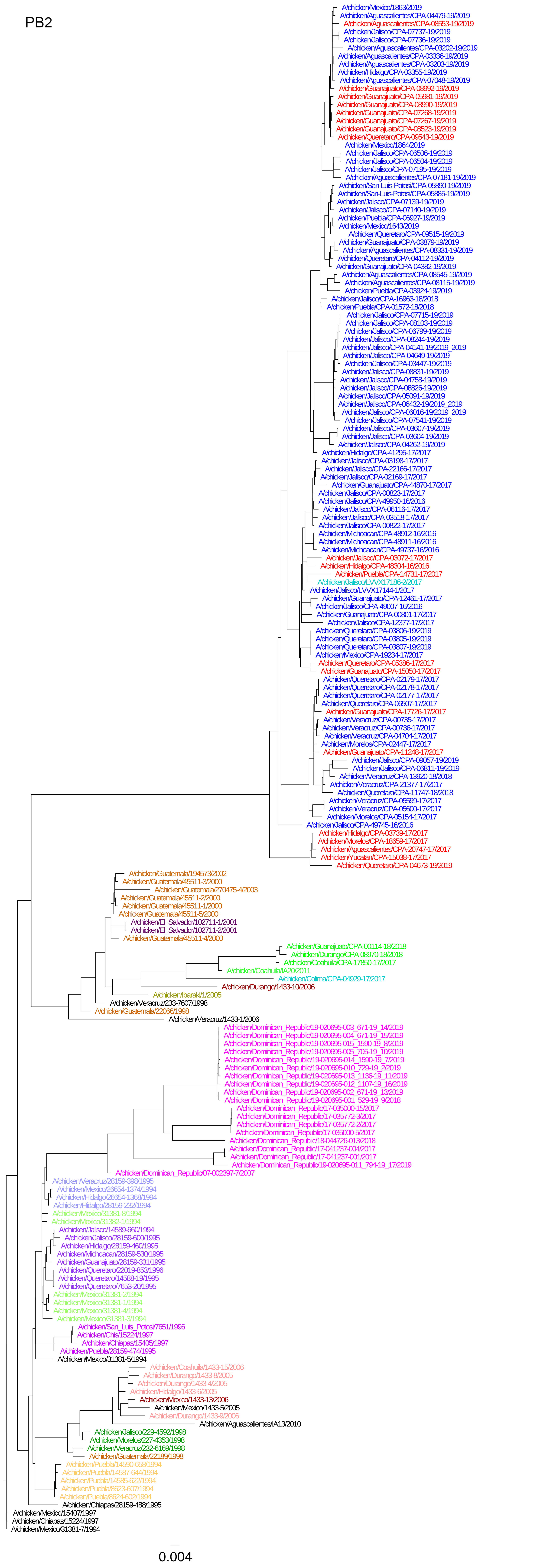
PB2
A/chicken/Mexico/1863/2019
A/chicken/Aguascalientes/CPA-04479-19/2019
A/chicken/Aguascalientes/CPA-08553-19/2019
A/chicken/Jalisco/CPA-07737-19/2019
A/chicken/Jalisco/CPA-07736-19/2019
A/chicken/Aguascalientes/CPA-03202-19/2019
A/chicken/Aguascalientes/CPA-03336-19/2019
A/chicken/Aguascalientes/CPA-03203-19/2019
A/chicken/Hidalgo/CPA-03355-19/2019
A/chicken/Aguascalientes/CPA-07048-19/2019
A/chicken/Guanajuato/CPA-08992-19/2019
A/chicken/Guanajuato/CPA-05981-19/2019
A/chicken/Guanajuato/CPA-08990-19/2019
A/chicken/Guanajuato/CPA-07268-19/2019
A/chicken/Guanajuato/CPA-07267-19/2019
A/chicken/Guanajuato/CPA-08523-19/2019
A/chicken/Queretaro/CPA-09543-19/2019
A/chicken/Mexico/1864/2019
A/chicken/Jalisco/CPA-06506-19/2019
A/chicken/Jalisco/CPA-06504-19/2019
A/chicken/Jalisco/CPA-07195-19/2019
A/chicken/Aguascalientes/CPA-07181-19/2019
A/chicken/San-Luis-Potosi/CPA-05890-19/2019
A/chicken/San-Luis-Potosi/CPA-05885-19/2019
A/chicken/Jalisco/CPA-07139-19/2019
A/chicken/Jalisco/CPA-07140-19/2019
A/chicken/Puebla/CPA-06927-19/2019
A/chicken/Mexico/1643/2019
A/chicken/Queretaro/CPA-09515-19/2019
A/chicken/Guanajuato/CPA-03879-19/2019
A/chicken/Aguascalientes/CPA-08331-19/2019
A/chicken/Queretaro/CPA-04112-19/2019
A/chicken/Guanajuato/CPA-04382-19/2019
A/chicken/Aguascalientes/CPA-08545-19/2019
A/chicken/Aguascalientes/CPA-08115-19/2019
A/chicken/Puebla/CPA-03924-19/2019
A/chicken/Jalisco/CPA-16963-18/2018
A/chicken/Puebla/CPA-01572-18/2018
A/chicken/Jalisco/CPA-07715-19/2019
A/chicken/Jalisco/CPA-08103-19/2019
A/chicken/Jalisco/CPA-06799-19/2019
A/chicken/Jalisco/CPA-08244-19/2019
A/chicken/Jalisco/CPA-04141-19/2019_2019
A/chicken/Jalisco/CPA-04649-19/2019
A/chicken/Jalisco/CPA-03447-19/2019
A/chicken/Jalisco/CPA-08831-19/2019
A/chicken/Jalisco/CPA-04758-19/2019
A/chicken/Jalisco/CPA-08826-19/2019
A/chicken/Jalisco/CPA-05091-19/2019
A/chicken/Jalisco/CPA-06432-19/2019_2019
A/chicken/Jalisco/CPA-06016-19/2019_2019
A/chicken/Jalisco/CPA-07541-19/2019
A/chicken/Jalisco/CPA-03607-19/2019
A/chicken/Jalisco/CPA-03604-19/2019
A/chicken/Jalisco/CPA-04262-19/2019
A/chicken/Hidalgo/CPA-41295-17/2017
A/chicken/Jalisco/CPA-03198-17/2017
A/chicken/Jalisco/CPA-22166-17/2017
A/chicken/Jalisco/CPA-02169-17/2017
A/chicken/Guanajuato/CPA-44870-17/2017
A/chicken/Jalisco/CPA-00823-17/2017
A/chicken/Jalisco/CPA-49950-16/2016
A/chicken/Jalisco/CPA-06116-17/2017
A/chicken/Jalisco/CPA-03518-17/2017
A/chicken/Jalisco/CPA-00822-17/2017
A/chicken/Michoacan/CPA-48912-16/2016
A/chicken/Michoacan/CPA-48911-16/2016
A/chicken/Michoacan/CPA-49737-16/2016
A/chicken/Jalisco/CPA-03072-17/2017
A/chicken/Hidalgo/CPA-48304-16/2016
A/chicken/Puebla/CPA-14731-17/2017
A/chicken/Jalisco/LVVX17186-2/2017
A/chicken/Jalisco/LVVX17144-1/2017
A/chicken/Guanajuato/CPA-12461-17/2017
A/chicken/Jalisco/CPA-49007-16/2016
A/chicken/Guanajuato/CPA-00801-17/2017
A/chicken/Jalisco/CPA-12377-17/2017
A/chicken/Queretaro/CPA-03806-19/2019
A/chicken/Queretaro/CPA-03805-19/2019
A/chicken/Queretaro/CPA-03807-19/2019
A/chicken/Mexico/CPA-19234-17/2017
A/chicken/Queretaro/CPA-05386-17/2017
A/chicken/Guanajuato/CPA-15050-17/2017
A/chicken/Queretaro/CPA-02179-17/2017
A/chicken/Queretaro/CPA-02178-17/2017
A/chicken/Queretaro/CPA-02177-17/2017
A/chicken/Queretaro/CPA-06507-17/2017
A/chicken/Guanajuato/CPA-17726-17/2017
A/chicken/Veracruz/CPA-00735-17/2017
A/chicken/Veracruz/CPA-00736-17/2017
A/chicken/Veracruz/CPA-04704-17/2017
A/chicken/Morelos/CPA-02447-17/2017
A/chicken/Guanajuato/CPA-11248-17/2017
A/chicken/Jalisco/CPA-09057-19/2019
A/chicken/Jalisco/CPA-06811-19/2019
A/chicken/Veracruz/CPA-13920-18/2018
A/chicken/Veracruz/CPA-21377-17/2017
A/chicken/Queretaro/CPA-11747-18/2018
A/chicken/Veracruz/CPA-05599-17/2017
A/chicken/Veracruz/CPA-05600-17/2017
A/chicken/Morelos/CPA-05154-17/2017
A/chicken/Jalisco/CPA-49745-16/2016
A/chicken/Hidalgo/CPA-03739-17/2017
A/chicken/Morelos/CPA-18659-17/2017
A/chicken/Aguascalientes/CPA-20747-17/2017
A/chicken/Yucatan/CPA-15038-17/2017
A/chicken/Queretaro/CPA-04673-19/2019
A/chicken/Guatemala/194573/2002
A/chicken/Guatemala/45511-3/2000
A/chicken/Guatemala/270475-4/2003
A/chicken/Guatemala/45511-2/2000
A/chicken/Guatemala/45511-1/2000
A/chicken/Guatemala/45511-5/2000
A/chicken/El_Salvador/102711-1/2001
A/chicken/El_Salvador/102711-2/2001
A/chicken/Guatemala/45511-4/2000
A/chicken/Guanajuato/CPA-00114-18/2018
A/chicken/Durango/CPA-08970-18/2018
A/chicken/Coahuila/CPA-17850-17/2017
A/chicken/Coahuila/IA20/2011
A/chicken/Colima/CPA-04929-17/2017
A/chicken/Durango/1433-10/2006
A/chicken/Ibaraki/1/2005
A/chicken/Veracruz/233-7607/1998
A/chicken/Guatemala/22066/1998
A/chicken/Veracruz/1433-1/2006
A/chicken/Dominican_Republic/19-020695-003_671-19_14/2019
A/chicken/Dominican_Republic/19-020695-004_671-19_15/2019
A/chicken/Dominican_Republic/19-020695-015_1590-19_8/2019
A/chicken/Dominican_Republic/19-020695-005_705-19_10/2019
A/chicken/Dominican_Republic/19-020695-014_1590-19_7/2019
A/chicken/Dominican_Republic/19-020695-010_729-19_2/2019
A/chicken/Dominican_Republic/19-020695-013_1136-19_11/2019
A/chicken/Dominican_Republic/19-020695-012_1107-19_16/2019
A/chicken/Dominican_Republic/19-020695-002_671-19_13/2019
A/chicken/Dominican_Republic/19-020695-001_529-19_9/2018
A/chicken/Dominican_Republic/17-035000-15/2017
A/chicken/Dominican_Republic/17-035772-3/2017
A/chicken/Dominican_Republic/17-035772-2/2017
A/chicken/Dominican_Republic/17-035000-5/2017
A/chicken/Dominican_Republic/18-044726-013/2018
A/chicken/Dominican_Republic/17-041237-004/2017
A/chicken/Dominican_Republic/17-041237-001/2017
A/chicken/Dominican_Republic/19-020695-011_794-19_17/2019
A/chicken/Dominican_Republic/07-002397-7/2007
A/chicken/Veracruz/28159-398/1995
A/chicken/Mexico/26654-1374/1994
A/chicken/Hidalgo/26654-1368/1994
A/chicken/Hidalgo/28159-232/1994
A/chicken/Mexico/31381-8/1994
A/chicken/Mexico/31382-1/1994
A/chicken/Jalisco/14589-660/1994
A/chicken/Jalisco/28159-600/1995
A/chicken/Hidalgo/28159-460/1995
A/chicken/Michoacan/28159-530/1995
A/chicken/Guanajuato/28159-331/1995
A/chicken/Queretaro/22019-853/1996
A/chicken/Queretaro/14588-19/1995
A/chicken/Queretaro/7653-20/1995
A/chicken/Mexico/31381-2/1994
A/chicken/Mexico/31381-1/1994
A/chicken/Mexico/31381-4/1994
A/chicken/Mexico/31381-3/1994
A/chicken/San_Luis_Potosi/7651/1996
A/chicken/Chis/15224/1997
A/chicken/Chiapas/15405/1997
A/chicken/Puebla/28159-474/1995
A/chicken/Mexico/31381-5/1994
A/chicken/Coahuila/1433-15/2006
A/chicken/Durango/1433-8/2005
A/chicken/Durango/1433-4/2005
A/chicken/Hidalgo/1433-6/2005
A/chicken/Mexico/1433-13/2006
A/chicken/Mexico/1433-5/2005
A/chicken/Durango/1433-9/2006
A/chicken/Aguascalientes/IA13/2010
A/chicken/Jalisco/229-4592/1998
A/chicken/Morelos/227-4353/1998
A/chicken/Veracruz/232-6169/1998
A/chicken/Guatemala/22189/1998
A/chicken/Puebla/14590-658/1994
A/chicken/Puebla/14587-644/1994
A/chicken/Puebla/14585-622/1994
A/chicken/Puebla/8623-607/1994
A/chicken/Puebla/8624-602/1994
A/chicken/Chiapas/28159-488/1995
A/chicken/Mexico/15407/1997
A/chicken/Chiapas/15224/1997
A/chicken/Mexico/31381-7/1994
0.004

PB1

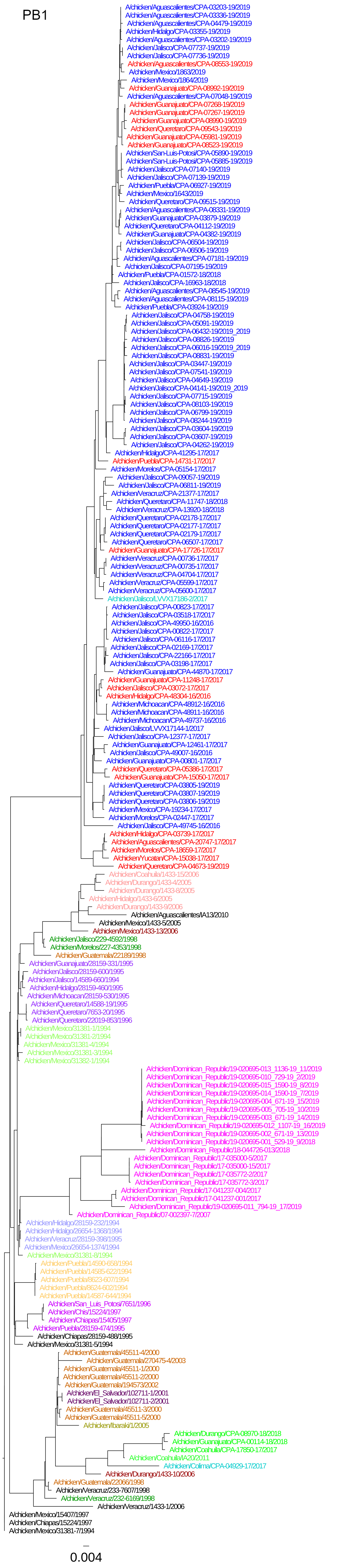

A/chicken/Aguascalientes/CPA-03203-19/2019
A/chicken/Aguascalientes/CPA-03336-19/2019
A/chicken/Aguascalientes/CPA-04479-19/2019
A/chicken/Hidalgo/CPA-03355-19/2019
A/chicken/Aguascalientes/CPA-03202-19/2019
A/chicken/Jalisco/CPA-07737-19/2019
A/chicken/Jalisco/CPA-07736-19/2019
A/chicken/Aguascalientes/CPA-08553-19/2019
A/chicken/Mexico/1863/2019
A/chicken/Mexico/1864/2019
A/chicken/Guanajuato/CPA-08992-19/2019
A/chicken/Aguascalientes/CPA-07048-19/2019
A/chicken/Guanajuato/CPA-07268-19/2019
A/chicken/Guanajuato/CPA-07267-19/2019
A/chicken/Guanajuato/CPA-08990-19/2019
A/chicken/Queretaro/CPA-09543-19/2019
A/chicken/Guanajuato/CPA-05981-19/2019
A/chicken/Guanajuato/CPA-08523-19/2019
A/chicken/San-Luis-Potosi/CPA-05890-19/2019
A/chicken/San-Luis-Potosi/CPA-05885-19/2019
A/chicken/Jalisco/CPA-07140-19/2019
A/chicken/Jalisco/CPA-07139-19/2019
A/chicken/Puebla/CPA-06927-19/2019
A/chicken/Mexico/1643/2019
A/chicken/Queretaro/CPA-09515-19/2019
A/chicken/Aguascalientes/CPA-08331-19/2019
A/chicken/Guanajuato/CPA-03879-19/2019
A/chicken/Queretaro/CPA-04112-19/2019
A/chicken/Guanajuato/CPA-04382-19/2019
A/chicken/Jalisco/CPA-06504-19/2019
A/chicken/Jalisco/CPA-06506-19/2019
A/chicken/Aguascalientes/CPA-07181-19/2019
A/chicken/Jalisco/CPA-07195-19/2019
A/chicken/Puebla/CPA-01572-18/2018
A/chicken/Jalisco/CPA-16963-18/2018
A/chicken/Aguascalientes/CPA-08545-19/2019
A/chicken/Aguascalientes/CPA-08115-19/2019
A/chicken/Puebla/CPA-03924-19/2019
A/chicken/Jalisco/CPA-04758-19/2019
A/chicken/Jalisco/CPA-05091-19/2019
A/chicken/Jalisco/CPA-06432-19/2019_2019
A/chicken/Jalisco/CPA-08826-19/2019
A/chicken/Jalisco/CPA-06016-19/2019_2019
A/chicken/Jalisco/CPA-08831-19/2019
A/chicken/Jalisco/CPA-03447-19/2019
A/chicken/Jalisco/CPA-07541-19/2019
A/chicken/Jalisco/CPA-04649-19/2019
A/chicken/Jalisco/CPA-04141-19/2019_2019
A/chicken/Jalisco/CPA-07715-19/2019
A/chicken/Jalisco/CPA-08103-19/2019
A/chicken/Jalisco/CPA-06799-19/2019
A/chicken/Jalisco/CPA-08244-19/2019
A/chicken/Jalisco/CPA-03604-19/2019
A/chicken/Jalisco/CPA-03607-19/2019
A/chicken/Jalisco/CPA-04262-19/2019
A/chicken/Hidalgo/CPA-41295-17/2017
A/chicken/Puebla/CPA-14731-17/2017
A/chicken/Morelos/CPA-05154-17/2017
A/chicken/Jalisco/CPA-09057-19/2019
A/chicken/Jalisco/CPA-06811-19/2019
A/chicken/Veracruz/CPA-21377-17/2017
A/chicken/Queretaro/CPA-11747-18/2018
A/chicken/Veracruz/CPA-13920-18/2018
A/chicken/Queretaro/CPA-02178-17/2017
A/chicken/Queretaro/CPA-02177-17/2017
A/chicken/Queretaro/CPA-02179-17/2017
A/chicken/Queretaro/CPA-06507-17/2017
A/chicken/Guanajuato/CPA-17726-17/2017
A/chicken/Veracruz/CPA-00736-17/2017
A/chicken/Veracruz/CPA-00735-17/2017
A/chicken/Veracruz/CPA-04704-17/2017
A/chicken/Veracruz/CPA-05599-17/2017
A/chicken/Veracruz/CPA-05600-17/2017
A/chicken/Jalisco/LVVX17186-2/2017
A/chicken/Jalisco/CPA-00823-17/2017
A/chicken/Jalisco/CPA-03518-17/2017
A/chicken/Jalisco/CPA-49950-16/2016
A/chicken/Jalisco/CPA-00822-17/2017
A/chicken/Jalisco/CPA-06116-17/2017
A/chicken/Jalisco/CPA-02169-17/2017
A/chicken/Jalisco/CPA-22166-17/2017
A/chicken/Jalisco/CPA-03198-17/2017
A/chicken/Guanajuato/CPA-44870-17/2017
A/chicken/Guanajuato/CPA-11248-17/2017
A/chicken/Jalisco/CPA-03072-17/2017
A/chicken/Hidalgo/CPA-48304-16/2016
A/chicken/Michoacan/CPA-48912-16/2016
A/chicken/Michoacan/CPA-48911-16/2016
A/chicken/Michoacan/CPA-49737-16/2016
A/chicken/Jalisco/LVVX17144-1/2017
A/chicken/Jalisco/CPA-12377-17/2017
A/chicken/Guanajuato/CPA-12461-17/2017
A/chicken/Jalisco/CPA-49007-16/2016
A/chicken/Guanajuato/CPA-00801-17/2017
A/chicken/Queretaro/CPA-05386-17/2017
A/chicken/Guanajuato/CPA-15050-17/2017
A/chicken/Queretaro/CPA-03805-19/2019
A/chicken/Queretaro/CPA-03807-19/2019
A/chicken/Queretaro/CPA-03806-19/2019
A/chicken/Mexico/CPA-19234-17/2017
A/chicken/Morelos/CPA-02447-17/2017
A/chicken/Jalisco/CPA-49745-16/2016
A/chicken/Hidalgo/CPA-03739-17/2017
A/chicken/Aguascalientes/CPA-20747-17/2017
A/chicken/Morelos/CPA-18659-17/2017
A/chicken/Yucatan/CPA-15038-17/2017
A/chicken/Queretaro/CPA-04673-19/2019
A/chicken/Coahuila/1433-15/2006
A/chicken/Durango/1433-4/2005
A/chicken/Durango/1433-8/2005
A/chicken/Hidalgo/1433-6/2005
A/chicken/Durango/1433-9/2006
A/chicken/Aguascalientes/IA13/2010
A/chicken/Mexico/1433-5/2005
A/chicken/Mexico/1433-13/2006
A/chicken/Jalisco/229-4592/1998
A/chicken/Morelos/227-4353/1998
A/chicken/Guatemala/22189/1998
A/chicken/Guanajuato/28159-331/1995
A/chicken/Jalisco/28159-600/1995
A/chicken/Jalisco/14589-660/1994
A/chicken/Hidalgo/28159-460/1995
A/chicken/Michoacan/28159-530/1995
A/chicken/Queretaro/14588-19/1995
A/chicken/Queretaro/7653-20/1995
A/chicken/Queretaro/22019-853/1996
A/chicken/Mexico/31381-1/1994
A/chicken/Mexico/31381-2/1994
A/chicken/Mexico/31381-4/1994
A/chicken/Mexico/31381-3/1994
A/chicken/Mexico/31382-1/1994
A/chicken/Dominican_Republic/19-020695-013_1136-19_11/2019
A/chicken/Dominican_Republic/19-020695-010_729-19_2/2019
A/chicken/Dominican_Republic/19-020695-015_1590-19_8/2019
A/chicken/Dominican_Republic/19-020695-014_1590-19_7/2019
A/chicken/Dominican_Republic/19-020695-004_671-19_15/2019
A/chicken/Dominican_Republic/19-020695-005_705-19_10/2019
A/chicken/Dominican_Republic/19-020695-003_671-19_14/2019
A/chicken/Dominican_Republic/19-020695-012_1107-19_16/2019
A/chicken/Dominican_Republic/19-020695-002_671-19_13/2019
A/chicken/Dominican_Republic/19-020695-001_529-19_9/2018
A/chicken/Dominican_Republic/18-044726-013/2018
A/chicken/Dominican_Republic/17-035000-5/2017
A/chicken/Dominican_Republic/17-035000-15/2017
A/chicken/Dominican_Republic/17-035772-2/2017
A/chicken/Dominican_Republic/17-035772-3/2017
A/chicken/Dominican_Republic/17-041237-004/2017
A/chicken/Dominican_Republic/17-041237-001/2017
A/chicken/Dominican_Republic/19-020695-011_794-19_17/2019
A/chicken/Dominican_Republic/07-002397-7/2007
A/chicken/Hidalgo/28159-232/1994
A/chicken/Hidalgo/26654-1368/1994
A/chicken/Veracruz/28159-398/1995
A/chicken/Mexico/26654-1374/1994
A/chicken/Mexico/31381-8/1994
A/chicken/Puebla/14590-658/1994
A/chicken/Puebla/14585-622/1994
A/chicken/Puebla/8623-607/1994
A/chicken/Puebla/8624-602/1994
A/chicken/Puebla/14587-644/1994
A/chicken/San_Luis_Potosi/7651/1996
A/chicken/Chis/15224/1997
A/chicken/Chiapas/15405/1997
A/chicken/Puebla/28159-474/1995
A/chicken/Chiapas/28159-488/1995
A/chicken/Mexico/31381-5/1994
A/chicken/Guatemala/45511-4/2000
A/chicken/Guatemala/270475-4/2003
A/chicken/Guatemala/45511-1/2000
A/chicken/Guatemala/45511-2/2000
A/chicken/Guatemala/194573/2002
A/chicken/El_Salvador/102711-1/2001
A/chicken/El_Salvador/102711-2/2001
A/chicken/Guatemala/45511-3/2000
A/chicken/Guatemala/45511-5/2000
A/chicken/Ibaraki/1/2005
A/chicken/Durango/CPA-08970-18/2018
A/chicken/Guanajuato/CPA-00114-18/2018
A/chicken/Coahuila/CPA-17850-17/2017
A/chicken/Coahuila/IA20/2011
A/chicken/Colima/CPA-04929-17/2017
A/chicken/Durango/1433-10/2006
A/chicken/Guatemala/22066/1998
A/chicken/Veracruz/233-7607/1998
A/chicken/Veracruz/232-6169/1998
A/chicken/Veracruz/1433-1/2006
A/chicken/Mexico/15407/1997
A/chicken/Chiapas/15224/1997
A/chicken/Mexico/31381-7/1994
0.004

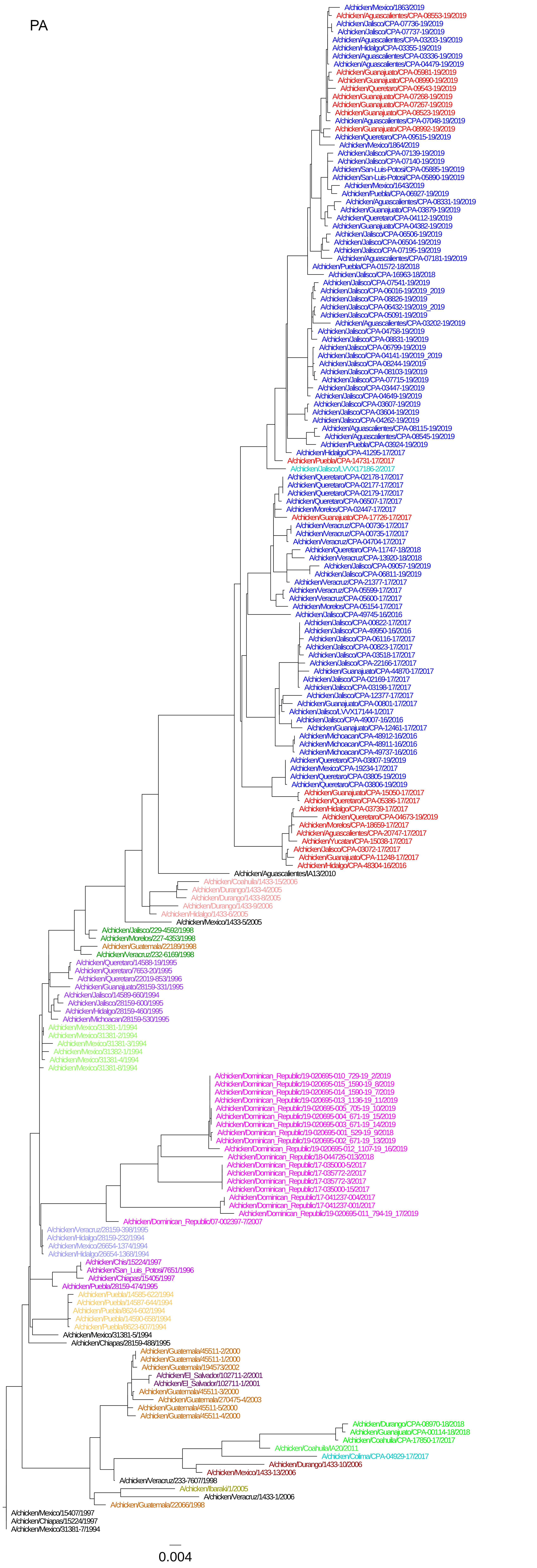
PA
A/chicken/Mexico/1863/2019
A/chicken/Aguascalientes/CPA-08553-19/2019
A/chicken/Jalisco/CPA-07736-19/2019
A/chicken/Jalisco/CPA-07737-19/2019
A/chicken/Aguascalientes/CPA-03203-19/2019
A/chicken/Hidalgo/CPA-03355-19/2019
A/chicken/Aguascalientes/CPA-03336-19/2019
A/chicken/Aguascalientes/CPA-04479-19/2019
A/chicken/Guanajuato/CPA-05981-19/2019
A/chicken/Guanajuato/CPA-08990-19/2019
A/chicken/Queretaro/CPA-09543-19/2019
A/chicken/Guanajuato/CPA-07268-19/2019
A/chicken/Guanajuato/CPA-07267-19/2019
A/chicken/Guanajuato/CPA-08523-19/2019
A/chicken/Aguascalientes/CPA-07048-19/2019
A/chicken/Guanajuato/CPA-08992-19/2019
A/chicken/Queretaro/CPA-09515-19/2019
A/chicken/Mexico/1864/2019
A/chicken/Jalisco/CPA-07139-19/2019
A/chicken/Jalisco/CPA-07140-19/2019
A/chicken/San-Luis-Potosi/CPA-05885-19/2019
A/chicken/San-Luis-Potosi/CPA-05890-19/2019
A/chicken/Mexico/1643/2019
A/chicken/Puebla/CPA-06927-19/2019
A/chicken/Aguascalientes/CPA-08331-19/2019
A/chicken/Guanajuato/CPA-03879-19/2019
A/chicken/Queretaro/CPA-04112-19/2019
A/chicken/Guanajuato/CPA-04382-19/2019
A/chicken/Jalisco/CPA-06506-19/2019
A/chicken/Jalisco/CPA-06504-19/2019
A/chicken/Jalisco/CPA-07195-19/2019
A/chicken/Aguascalientes/CPA-07181-19/2019
A/chicken/Puebla/CPA-01572-18/2018
A/chicken/Jalisco/CPA-16963-18/2018
A/chicken/Jalisco/CPA-07541-19/2019
A/chicken/Jalisco/CPA-06016-19/2019_2019
A/chicken/Jalisco/CPA-08826-19/2019
A/chicken/Jalisco/CPA-06432-19/2019_2019
A/chicken/Jalisco/CPA-05091-19/2019
A/chicken/Aguascalientes/CPA-03202-19/2019
A/chicken/Jalisco/CPA-04758-19/2019
A/chicken/Jalisco/CPA-08831-19/2019
A/chicken/Jalisco/CPA-06799-19/2019
A/chicken/Jalisco/CPA-04141-19/2019_2019
A/chicken/Jalisco/CPA-08244-19/2019
A/chicken/Jalisco/CPA-08103-19/2019
A/chicken/Jalisco/CPA-07715-19/2019
A/chicken/Jalisco/CPA-03447-19/2019
A/chicken/Jalisco/CPA-04649-19/2019
A/chicken/Jalisco/CPA-03607-19/2019
A/chicken/Jalisco/CPA-03604-19/2019
A/chicken/Jalisco/CPA-04262-19/2019
A/chicken/Aguascalientes/CPA-08115-19/2019
A/chicken/Aguascalientes/CPA-08545-19/2019
A/chicken/Puebla/CPA-03924-19/2019
A/chicken/Hidalgo/CPA-41295-17/2017
A/chicken/Puebla/CPA-14731-17/2017
A/chicken/Jalisco/LVVX17186-2/2017
A/chicken/Queretaro/CPA-02178-17/2017
A/chicken/Queretaro/CPA-02177-17/2017
A/chicken/Queretaro/CPA-02179-17/2017
A/chicken/Queretaro/CPA-06507-17/2017
A/chicken/Morelos/CPA-02447-17/2017
A/chicken/Guanajuato/CPA-17726-17/2017
A/chicken/Veracruz/CPA-00736-17/2017
A/chicken/Veracruz/CPA-00735-17/2017
A/chicken/Veracruz/CPA-04704-17/2017
A/chicken/Queretaro/CPA-11747-18/2018
A/chicken/Veracruz/CPA-13920-18/2018
A/chicken/Jalisco/CPA-09057-19/2019
A/chicken/Jalisco/CPA-06811-19/2019
A/chicken/Veracruz/CPA-21377-17/2017
A/chicken/Veracruz/CPA-05599-17/2017
A/chicken/Veracruz/CPA-05600-17/2017
A/chicken/Morelos/CPA-05154-17/2017
A/chicken/Jalisco/CPA-49745-16/2016
A/chicken/Jalisco/CPA-00822-17/2017
A/chicken/Jalisco/CPA-49950-16/2016
A/chicken/Jalisco/CPA-06116-17/2017
A/chicken/Jalisco/CPA-00823-17/2017
A/chicken/Jalisco/CPA-03518-17/2017
A/chicken/Jalisco/CPA-22166-17/2017
A/chicken/Guanajuato/CPA-44870-17/2017
A/chicken/Jalisco/CPA-02169-17/2017
A/chicken/Jalisco/CPA-03198-17/2017
A/chicken/Jalisco/CPA-12377-17/2017
A/chicken/Guanajuato/CPA-00801-17/2017
A/chicken/Jalisco/LVVX17144-1/2017
A/chicken/Jalisco/CPA-49007-16/2016
A/chicken/Guanajuato/CPA-12461-17/2017
A/chicken/Michoacan/CPA-48912-16/2016
A/chicken/Michoacan/CPA-48911-16/2016
A/chicken/Michoacan/CPA-49737-16/2016
A/chicken/Queretaro/CPA-03807-19/2019
A/chicken/Mexico/CPA-19234-17/2017
A/chicken/Queretaro/CPA-03805-19/2019
A/chicken/Queretaro/CPA-03806-19/2019
A/chicken/Guanajuato/CPA-15050-17/2017
A/chicken/Queretaro/CPA-05386-17/2017
A/chicken/Hidalgo/CPA-03739-17/2017
A/chicken/Queretaro/CPA-04673-19/2019
A/chicken/Morelos/CPA-18659-17/2017
A/chicken/Aguascalientes/CPA-20747-17/2017
A/chicken/Yucatan/CPA-15038-17/2017
A/chicken/Jalisco/CPA-03072-17/2017
A/chicken/Guanajuato/CPA-11248-17/2017
A/chicken/Hidalgo/CPA-48304-16/2016
A/chicken/Aguascalientes/IA13/2010
A/chicken/Coahuila/1433-15/2006
A/chicken/Durango/1433-4/2005
A/chicken/Durango/1433-8/2005
A/chicken/Durango/1433-9/2006
A/chicken/Hidalgo/1433-6/2005
A/chicken/Mexico/1433-5/2005
A/chicken/Jalisco/229-4592/1998
A/chicken/Morelos/227-4353/1998
A/chicken/Guatemala/22189/1998
A/chicken/Veracruz/232-6169/1998
A/chicken/Queretaro/14588-19/1995
A/chicken/Queretaro/7653-20/1995
A/chicken/Queretaro/22019-853/1996
A/chicken/Guanajuato/28159-331/1995
A/chicken/Jalisco/14589-660/1994
A/chicken/Jalisco/28159-600/1995
A/chicken/Hidalgo/28159-460/1995
A/chicken/Michoacan/28159-530/1995
A/chicken/Mexico/31381-1/1994
A/chicken/Mexico/31381-2/1994
A/chicken/Mexico/31381-3/1994
A/chicken/Mexico/31382-1/1994
A/chicken/Mexico/31381-4/1994
A/chicken/Mexico/31381-8/1994
A/chicken/Dominican_Republic/19-020695-010_729-19_2/2019
A/chicken/Dominican_Republic/19-020695-015_1590-19_8/2019
A/chicken/Dominican_Republic/19-020695-014_1590-19_7/2019
A/chicken/Dominican_Republic/19-020695-013_1136-19_11/2019
A/chicken/Dominican_Republic/19-020695-005_705-19_10/2019
A/chicken/Dominican_Republic/19-020695-004_671-19_15/2019
A/chicken/Dominican_Republic/19-020695-003_671-19_14/2019
A/chicken/Dominican_Republic/19-020695-001_529-19_9/2018
A/chicken/Dominican_Republic/19-020695-002_671-19_13/2019
A/chicken/Dominican_Republic/19-020695-012_1107-19_16/2019
A/chicken/Dominican_Republic/18-044726-013/2018
A/chicken/Dominican_Republic/17-035000-5/2017
A/chicken/Dominican_Republic/17-035772-2/2017
A/chicken/Dominican_Republic/17-035772-3/2017
A/chicken/Dominican_Republic/17-035000-15/2017
A/chicken/Dominican_Republic/17-041237-004/2017
A/chicken/Dominican_Republic/17-041237-001/2017
A/chicken/Dominican_Republic/19-020695-011_794-19_17/2019
A/chicken/Dominican_Republic/07-002397-7/2007
A/chicken/Veracruz/28159-398/1995
A/chicken/Hidalgo/28159-232/1994
A/chicken/Mexico/26654-1374/1994
A/chicken/Hidalgo/26654-1368/1994
A/chicken/Chis/15224/1997
A/chicken/San_Luis_Potosi/7651/1996
A/chicken/Chiapas/15405/1997
A/chicken/Puebla/28159-474/1995
A/chicken/Puebla/14585-622/1994
A/chicken/Puebla/14587-644/1994
A/chicken/Puebla/8624-602/1994
A/chicken/Puebla/14590-658/1994
A/chicken/Puebla/8623-607/1994
A/chicken/Mexico/31381-5/1994
A/chicken/Chiapas/28159-488/1995
A/chicken/Guatemala/45511-2/2000
A/chicken/Guatemala/45511-1/2000
A/chicken/Guatemala/194573/2002
A/chicken/El_Salvador/102711-2/2001
A/chicken/El_Salvador/102711-1/2001
A/chicken/Guatemala/45511-3/2000
A/chicken/Guatemala/270475-4/2003
A/chicken/Guatemala/45511-5/2000
A/chicken/Guatemala/45511-4/2000
A/chicken/Durango/CPA-08970-18/2018
A/chicken/Guanajuato/CPA-00114-18/2018
A/chicken/Coahuila/CPA-17850-17/2017
A/chicken/Coahuila/IA20/2011
A/chicken/Colima/CPA-04929-17/2017
A/chicken/Durango/1433-10/2006
A/chicken/Mexico/1433-13/2006
A/chicken/Veracruz/233-7607/1998
A/chicken/Ibaraki/1/2005
A/chicken/Veracruz/1433-1/2006
A/chicken/Guatemala/22066/1998
A/chicken/Mexico/15407/1997
A/chicken/Chiapas/15224/1997
A/chicken/Mexico/31381-7/1994
0.004

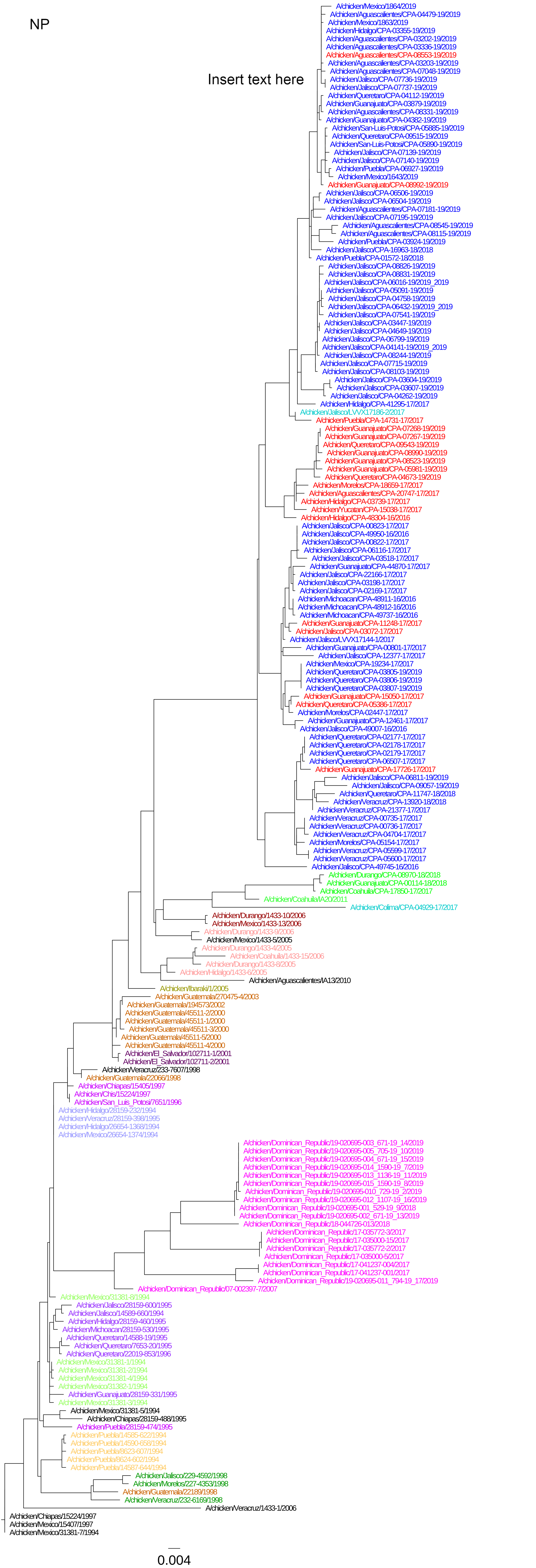
NP
Insert text here
A/chicken/Mexico/1864/2019
A/chicken/Aguascalientes/CPA-04479-19/2019
A/chicken/Mexico/1863/2019
A/chicken/Hidalgo/CPA-03355-19/2019
A/chicken/Aguascalientes/CPA-03202-19/2019
A/chicken/Aguascalientes/CPA-03336-19/2019
A/chicken/Aguascalientes/CPA-08553-19/2019
A/chicken/Aguascalientes/CPA-03203-19/2019
A/chicken/Aguascalientes/CPA-07048-19/2019
A/chicken/Jalisco/CPA-07736-19/2019
A/chicken/Jalisco/CPA-07737-19/2019
A/chicken/Queretaro/CPA-04112-19/2019
A/chicken/Guanajuato/CPA-03879-19/2019
A/chicken/Aguascalientes/CPA-08331-19/2019
A/chicken/Guanajuato/CPA-04382-19/2019
A/chicken/San-Luis-Potosi/CPA-05885-19/2019
A/chicken/Queretaro/CPA-09515-19/2019
A/chicken/San-Luis-Potosi/CPA-05890-19/2019
A/chicken/Jalisco/CPA-07139-19/2019
A/chicken/Jalisco/CPA-07140-19/2019
A/chicken/Puebla/CPA-06927-19/2019
A/chicken/Mexico/1643/2019
A/chicken/Guanajuato/CPA-08992-19/2019
A/chicken/Jalisco/CPA-06506-19/2019
A/chicken/Jalisco/CPA-06504-19/2019
A/chicken/Aguascalientes/CPA-07181-19/2019
A/chicken/Jalisco/CPA-07195-19/2019
A/chicken/Aguascalientes/CPA-08545-19/2019
A/chicken/Aguascalientes/CPA-08115-19/2019
A/chicken/Puebla/CPA-03924-19/2019
A/chicken/Jalisco/CPA-16963-18/2018
A/chicken/Puebla/CPA-01572-18/2018
A/chicken/Jalisco/CPA-08826-19/2019
A/chicken/Jalisco/CPA-08831-19/2019
A/chicken/Jalisco/CPA-06016-19/2019_2019
A/chicken/Jalisco/CPA-05091-19/2019
A/chicken/Jalisco/CPA-04758-19/2019
A/chicken/Jalisco/CPA-06432-19/2019_2019
A/chicken/Jalisco/CPA-07541-19/2019
A/chicken/Jalisco/CPA-03447-19/2019
A/chicken/Jalisco/CPA-04649-19/2019
A/chicken/Jalisco/CPA-06799-19/2019
A/chicken/Jalisco/CPA-04141-19/2019_2019
A/chicken/Jalisco/CPA-08244-19/2019
A/chicken/Jalisco/CPA-07715-19/2019
A/chicken/Jalisco/CPA-08103-19/2019
A/chicken/Jalisco/CPA-03604-19/2019
A/chicken/Jalisco/CPA-03607-19/2019
A/chicken/Jalisco/CPA-04262-19/2019
A/chicken/Hidalgo/CPA-41295-17/2017
A/chicken/Jalisco/LVVX17186-2/2017
A/chicken/Puebla/CPA-14731-17/2017
A/chicken/Guanajuato/CPA-07268-19/2019
A/chicken/Guanajuato/CPA-07267-19/2019
A/chicken/Queretaro/CPA-09543-19/2019
A/chicken/Guanajuato/CPA-08990-19/2019
A/chicken/Guanajuato/CPA-08523-19/2019
A/chicken/Guanajuato/CPA-05981-19/2019
A/chicken/Queretaro/CPA-04673-19/2019
A/chicken/Morelos/CPA-18659-17/2017
A/chicken/Aguascalientes/CPA-20747-17/2017
A/chicken/Hidalgo/CPA-03739-17/2017
A/chicken/Yucatan/CPA-15038-17/2017
A/chicken/Hidalgo/CPA-48304-16/2016
A/chicken/Jalisco/CPA-00823-17/2017
A/chicken/Jalisco/CPA-49950-16/2016
A/chicken/Jalisco/CPA-00822-17/2017
A/chicken/Jalisco/CPA-06116-17/2017
A/chicken/Jalisco/CPA-03518-17/2017
A/chicken/Guanajuato/CPA-44870-17/2017
A/chicken/Jalisco/CPA-22166-17/2017
A/chicken/Jalisco/CPA-03198-17/2017
A/chicken/Jalisco/CPA-02169-17/2017
A/chicken/Michoacan/CPA-48911-16/2016
A/chicken/Michoacan/CPA-48912-16/2016
A/chicken/Michoacan/CPA-49737-16/2016
A/chicken/Guanajuato/CPA-11248-17/2017
A/chicken/Jalisco/CPA-03072-17/2017
A/chicken/Jalisco/LVVX17144-1/2017
A/chicken/Guanajuato/CPA-00801-17/2017
A/chicken/Jalisco/CPA-12377-17/2017
A/chicken/Mexico/CPA-19234-17/2017
A/chicken/Queretaro/CPA-03805-19/2019
A/chicken/Queretaro/CPA-03806-19/2019
A/chicken/Queretaro/CPA-03807-19/2019
A/chicken/Guanajuato/CPA-15050-17/2017
A/chicken/Queretaro/CPA-05386-17/2017
A/chicken/Morelos/CPA-02447-17/2017
A/chicken/Guanajuato/CPA-12461-17/2017
A/chicken/Jalisco/CPA-49007-16/2016
A/chicken/Queretaro/CPA-02177-17/2017
A/chicken/Queretaro/CPA-02178-17/2017
A/chicken/Queretaro/CPA-02179-17/2017
A/chicken/Queretaro/CPA-06507-17/2017
A/chicken/Guanajuato/CPA-17726-17/2017
A/chicken/Jalisco/CPA-06811-19/2019
A/chicken/Jalisco/CPA-09057-19/2019
A/chicken/Queretaro/CPA-11747-18/2018
A/chicken/Veracruz/CPA-13920-18/2018
A/chicken/Veracruz/CPA-21377-17/2017
A/chicken/Veracruz/CPA-00735-17/2017
A/chicken/Veracruz/CPA-00736-17/2017
A/chicken/Veracruz/CPA-04704-17/2017
A/chicken/Morelos/CPA-05154-17/2017
A/chicken/Veracruz/CPA-05599-17/2017
A/chicken/Veracruz/CPA-05600-17/2017
A/chicken/Jalisco/CPA-49745-16/2016
A/chicken/Durango/CPA-08970-18/2018
A/chicken/Guanajuato/CPA-00114-18/2018
A/chicken/Coahuila/CPA-17850-17/2017
A/chicken/Coahuila/IA20/2011
A/chicken/Colima/CPA-04929-17/2017
A/chicken/Durango/1433-10/2006
A/chicken/Mexico/1433-13/2006
A/chicken/Durango/1433-9/2006
A/chicken/Mexico/1433-5/2005
A/chicken/Durango/1433-4/2005
A/chicken/Coahuila/1433-15/2006
A/chicken/Durango/1433-8/2005
A/chicken/Hidalgo/1433-6/2005
A/chicken/Aguascalientes/IA13/2010
A/chicken/Ibaraki/1/2005
A/chicken/Guatemala/270475-4/2003
A/chicken/Guatemala/194573/2002
A/chicken/Guatemala/45511-2/2000
A/chicken/Guatemala/45511-1/2000
A/chicken/Guatemala/45511-3/2000
A/chicken/Guatemala/45511-5/2000
A/chicken/Guatemala/45511-4/2000
A/chicken/El_Salvador/102711-1/2001
A/chicken/El_Salvador/102711-2/2001
A/chicken/Veracruz/233-7607/1998
A/chicken/Guatemala/22066/1998
A/chicken/Chiapas/15405/1997
A/chicken/Chis/15224/1997
A/chicken/San_Luis_Potosi/7651/1996
A/chicken/Hidalgo/28159-232/1994
A/chicken/Veracruz/28159-398/1995
A/chicken/Hidalgo/26654-1368/1994
A/chicken/Mexico/26654-1374/1994
A/chicken/Dominican_Republic/19-020695-003_671-19_14/2019
A/chicken/Dominican_Republic/19-020695-005_705-19_10/2019
A/chicken/Dominican_Republic/19-020695-004_671-19_15/2019
A/chicken/Dominican_Republic/19-020695-014_1590-19_7/2019
A/chicken/Dominican_Republic/19-020695-013_1136-19_11/2019
A/chicken/Dominican_Republic/19-020695-015_1590-19_8/2019
A/chicken/Dominican_Republic/19-020695-010_729-19_2/2019
A/chicken/Dominican_Republic/19-020695-012_1107-19_16/2019
A/chicken/Dominican_Republic/19-020695-001_529-19_9/2018
A/chicken/Dominican_Republic/19-020695-002_671-19_13/2019
A/chicken/Dominican_Republic/18-044726-013/2018
A/chicken/Dominican_Republic/17-035772-3/2017
A/chicken/Dominican_Republic/17-035000-15/2017
A/chicken/Dominican_Republic/17-035772-2/2017
A/chicken/Dominican_Republic/17-035000-5/2017
A/chicken/Dominican_Republic/17-041237-004/2017
A/chicken/Dominican_Republic/17-041237-001/2017
A/chicken/Dominican_Republic/19-020695-011_794-19_17/2019
A/chicken/Dominican_Republic/07-002397-7/2007
A/chicken/Mexico/31381-8/1994
A/chicken/Jalisco/28159-600/1995
A/chicken/Jalisco/14589-660/1994
A/chicken/Hidalgo/28159-460/1995
A/chicken/Michoacan/28159-530/1995
A/chicken/Queretaro/14588-19/1995
A/chicken/Queretaro/7653-20/1995
A/chicken/Queretaro/22019-853/1996
A/chicken/Mexico/31381-1/1994
A/chicken/Mexico/31381-2/1994
A/chicken/Mexico/31381-4/1994
A/chicken/Mexico/31382-1/1994
A/chicken/Guanajuato/28159-331/1995
A/chicken/Mexico/31381-3/1994
A/chicken/Mexico/31381-5/1994
A/chicken/Chiapas/28159-488/1995
A/chicken/Puebla/28159-474/1995
A/chicken/Puebla/14585-622/1994
A/chicken/Puebla/14590-658/1994
A/chicken/Puebla/8623-607/1994
A/chicken/Puebla/8624-602/1994
A/chicken/Puebla/14587-644/1994
A/chicken/Jalisco/229-4592/1998
A/chicken/Morelos/227-4353/1998
A/chicken/Guatemala/22189/1998
A/chicken/Veracruz/232-6169/1998
A/chicken/Veracruz/1433-1/2006
A/chicken/Chiapas/15224/1997
A/chicken/Mexico/15407/1997
A/chicken/Mexico/31381-7/1994
0.004

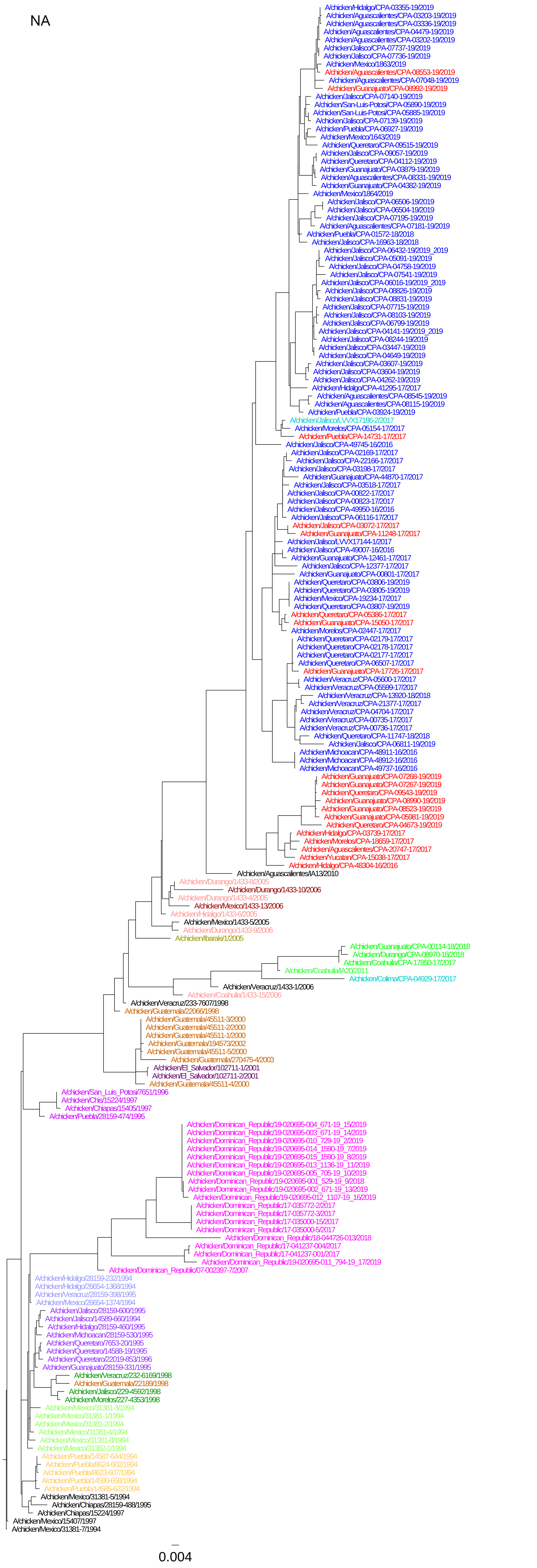
NA
A/chicken/Hidalgo/CPA-03355-19/2019
A/chicken/Aguascalientes/CPA-03203-19/2019
A/chicken/Aguascalientes/CPA-03336-19/2019
A/chicken/Aguascalientes/CPA-04479-19/2019
A/chicken/Aguascalientes/CPA-03202-19/2019
A/chicken/Jalisco/CPA-07737-19/2019
A/chicken/Jalisco/CPA-07736-19/2019
A/chicken/Mexico/1863/2019
A/chicken/Aguascalientes/CPA-08553-19/2019
A/chicken/Aguascalientes/CPA-07048-19/2019
A/chicken/Guanajuato/CPA-08992-19/2019
A/chicken/Jalisco/CPA-07140-19/2019
A/chicken/San-Luis-Potosi/CPA-05890-19/2019
A/chicken/San-Luis-Potosi/CPA-05885-19/2019
A/chicken/Jalisco/CPA-07139-19/2019
A/chicken/Puebla/CPA-06927-19/2019
A/chicken/Mexico/1643/2019
A/chicken/Queretaro/CPA-09515-19/2019
A/chicken/Jalisco/CPA-09057-19/2019
A/chicken/Queretaro/CPA-04112-19/2019
A/chicken/Guanajuato/CPA-03879-19/2019
A/chicken/Aguascalientes/CPA-08331-19/2019
A/chicken/Guanajuato/CPA-04382-19/2019
A/chicken/Mexico/1864/2019
A/chicken/Jalisco/CPA-06506-19/2019
A/chicken/Jalisco/CPA-06504-19/2019
A/chicken/Jalisco/CPA-07195-19/2019
A/chicken/Aguascalientes/CPA-07181-19/2019
A/chicken/Puebla/CPA-01572-18/2018
A/chicken/Jalisco/CPA-16963-18/2018
A/chicken/Jalisco/CPA-06432-19/2019_2019
A/chicken/Jalisco/CPA-05091-19/2019
A/chicken/Jalisco/CPA-04758-19/2019
A/chicken/Jalisco/CPA-07541-19/2019
A/chicken/Jalisco/CPA-06016-19/2019_2019
A/chicken/Jalisco/CPA-08826-19/2019
A/chicken/Jalisco/CPA-08831-19/2019
A/chicken/Jalisco/CPA-07715-19/2019
A/chicken/Jalisco/CPA-08103-19/2019
A/chicken/Jalisco/CPA-06799-19/2019
A/chicken/Jalisco/CPA-04141-19/2019_2019
A/chicken/Jalisco/CPA-08244-19/2019
A/chicken/Jalisco/CPA-03447-19/2019
A/chicken/Jalisco/CPA-04649-19/2019
A/chicken/Jalisco/CPA-03607-19/2019
A/chicken/Jalisco/CPA-03604-19/2019
A/chicken/Jalisco/CPA-04262-19/2019
A/chicken/Hidalgo/CPA-41295-17/2017
A/chicken/Aguascalientes/CPA-08545-19/2019
A/chicken/Aguascalientes/CPA-08115-19/2019
A/chicken/Puebla/CPA-03924-19/2019
A/chicken/Jalisco/LVVX17186-2/2017
A/chicken/Morelos/CPA-05154-17/2017
A/chicken/Puebla/CPA-14731-17/2017
A/chicken/Jalisco/CPA-49745-16/2016
A/chicken/Jalisco/CPA-02169-17/2017
A/chicken/Jalisco/CPA-22166-17/2017
A/chicken/Jalisco/CPA-03198-17/2017
A/chicken/Guanajuato/CPA-44870-17/2017
A/chicken/Jalisco/CPA-03518-17/2017
A/chicken/Jalisco/CPA-00822-17/2017
A/chicken/Jalisco/CPA-00823-17/2017
A/chicken/Jalisco/CPA-49950-16/2016
A/chicken/Jalisco/CPA-06116-17/2017
A/chicken/Jalisco/CPA-03072-17/2017
A/chicken/Guanajuato/CPA-11248-17/2017
A/chicken/Jalisco/LVVX17144-1/2017
A/chicken/Jalisco/CPA-49007-16/2016
A/chicken/Guanajuato/CPA-12461-17/2017
A/chicken/Jalisco/CPA-12377-17/2017
A/chicken/Guanajuato/CPA-00801-17/2017
A/chicken/Queretaro/CPA-03806-19/2019
A/chicken/Queretaro/CPA-03805-19/2019
A/chicken/Mexico/CPA-19234-17/2017
A/chicken/Queretaro/CPA-03807-19/2019
A/chicken/Queretaro/CPA-05386-17/2017
A/chicken/Guanajuato/CPA-15050-17/2017
A/chicken/Morelos/CPA-02447-17/2017
A/chicken/Queretaro/CPA-02179-17/2017
A/chicken/Queretaro/CPA-02178-17/2017
A/chicken/Queretaro/CPA-02177-17/2017
A/chicken/Queretaro/CPA-06507-17/2017
A/chicken/Guanajuato/CPA-17726-17/2017
A/chicken/Veracruz/CPA-05600-17/2017
A/chicken/Veracruz/CPA-05599-17/2017
A/chicken/Veracruz/CPA-13920-18/2018
A/chicken/Veracruz/CPA-21377-17/2017
A/chicken/Veracruz/CPA-04704-17/2017
A/chicken/Veracruz/CPA-00735-17/2017
A/chicken/Veracruz/CPA-00736-17/2017
A/chicken/Queretaro/CPA-11747-18/2018
A/chicken/Jalisco/CPA-06811-19/2019
A/chicken/Michoacan/CPA-48911-16/2016
A/chicken/Michoacan/CPA-48912-16/2016
A/chicken/Michoacan/CPA-49737-16/2016
A/chicken/Guanajuato/CPA-07268-19/2019
A/chicken/Guanajuato/CPA-07267-19/2019
A/chicken/Queretaro/CPA-09543-19/2019
A/chicken/Guanajuato/CPA-08990-19/2019
A/chicken/Guanajuato/CPA-08523-19/2019
A/chicken/Guanajuato/CPA-05981-19/2019
A/chicken/Queretaro/CPA-04673-19/2019
A/chicken/Hidalgo/CPA-03739-17/2017
A/chicken/Morelos/CPA-18659-17/2017
A/chicken/Aguascalientes/CPA-20747-17/2017
A/chicken/Yucatan/CPA-15038-17/2017
A/chicken/Hidalgo/CPA-48304-16/2016
A/chicken/Aguascalientes/IA13/2010
A/chicken/Durango/1433-8/2005
A/chicken/Durango/1433-10/2006
A/chicken/Durango/1433-4/2005
A/chicken/Mexico/1433-13/2006
A/chicken/Hidalgo/1433-6/2005
A/chicken/Mexico/1433-5/2005
A/chicken/Durango/1433-9/2006
A/chicken/Ibaraki/1/2005
A/chicken/Guanajuato/CPA-00114-18/2018
A/chicken/Durango/CPA-08970-18/2018
A/chicken/Coahuila/CPA-17850-17/2017
A/chicken/Coahuila/IA20/2011
A/chicken/Colima/CPA-04929-17/2017
A/chicken/Veracruz/1433-1/2006
A/chicken/Coahuila/1433-15/2006
A/chicken/Veracruz/233-7607/1998
A/chicken/Guatemala/22066/1998
A/chicken/Guatemala/45511-3/2000
A/chicken/Guatemala/45511-2/2000
A/chicken/Guatemala/45511-1/2000
A/chicken/Guatemala/194573/2002
A/chicken/Guatemala/45511-5/2000
A/chicken/Guatemala/270475-4/2003
A/chicken/El_Salvador/102711-1/2001
A/chicken/El_Salvador/102711-2/2001
A/chicken/Guatemala/45511-4/2000
A/chicken/San_Luis_Potosi/7651/1996
A/chicken/Chis/15224/1997
A/chicken/Chiapas/15405/1997
A/chicken/Puebla/28159-474/1995
A/chicken/Dominican_Republic/19-020695-004_671-19_15/2019
A/chicken/Dominican_Republic/19-020695-003_671-19_14/2019
A/chicken/Dominican_Republic/19-020695-010_729-19_2/2019
A/chicken/Dominican_Republic/19-020695-014_1590-19_7/2019
A/chicken/Dominican_Republic/19-020695-015_1590-19_8/2019
A/chicken/Dominican_Republic/19-020695-013_1136-19_11/2019
A/chicken/Dominican_Republic/19-020695-005_705-19_10/2019
A/chicken/Dominican_Republic/19-020695-001_529-19_9/2018
A/chicken/Dominican_Republic/19-020695-002_671-19_13/2019
A/chicken/Dominican_Republic/19-020695-012_1107-19_16/2019
A/chicken/Dominican_Republic/17-035772-2/2017
A/chicken/Dominican_Republic/17-035772-3/2017
A/chicken/Dominican_Republic/17-035000-15/2017
A/chicken/Dominican_Republic/17-035000-5/2017
A/chicken/Dominican_Republic/18-044726-013/2018
A/chicken/Dominican_Republic/17-041237-004/2017
A/chicken/Dominican_Republic/17-041237-001/2017
A/chicken/Dominican_Republic/19-020695-011_794-19_17/2019
A/chicken/Dominican_Republic/07-002397-7/2007
A/chicken/Hidalgo/28159-232/1994
A/chicken/Hidalgo/26654-1368/1994
A/chicken/Veracruz/28159-398/1995
A/chicken/Mexico/26654-1374/1994
A/chicken/Jalisco/28159-600/1995
A/chicken/Jalisco/14589-660/1994
A/chicken/Hidalgo/28159-460/1995
A/chicken/Michoacan/28159-530/1995
A/chicken/Queretaro/7653-20/1995
A/chicken/Queretaro/14588-19/1995
A/chicken/Queretaro/22019-853/1996
A/chicken/Guanajuato/28159-331/1995
A/chicken/Veracruz/232-6169/1998
A/chicken/Guatemala/22189/1998
A/chicken/Jalisco/229-4592/1998
A/chicken/Morelos/227-4353/1998
A/chicken/Mexico/31381-3/1994
A/chicken/Mexico/31381-1/1994
A/chicken/Mexico/31381-2/1994
A/chicken/Mexico/31381-4/1994
A/chicken/Mexico/31381-8/1994
A/chicken/Mexico/31382-1/1994
A/chicken/Puebla/14587-644/1994
A/chicken/Puebla/8624-602/1994
A/chicken/Puebla/8623-607/1994
A/chicken/Puebla/14590-658/1994
A/chicken/Puebla/14585-622/1994
A/chicken/Mexico/31381-5/1994
A/chicken/Chiapas/28159-488/1995
A/chicken/Chiapas/15224/1997
A/chicken/Mexico/15407/1997
A/chicken/Mexico/31381-7/1994
0.004

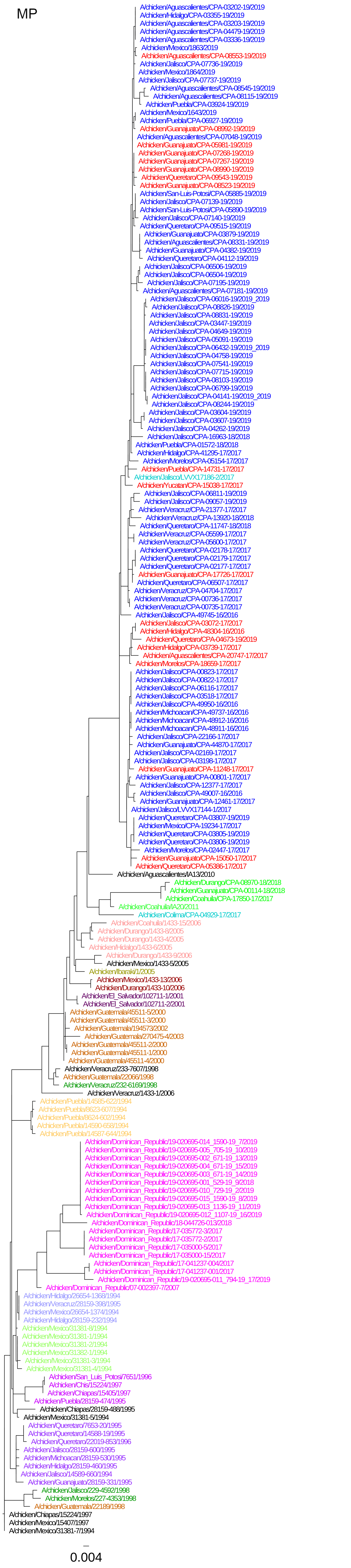
MP
A/chicken/Aguascalientes/CPA-03202-19/2019
A/chicken/Hidalgo/CPA-03355-19/2019
A/chicken/Aguascalientes/CPA-03203-19/2019
A/chicken/Aguascalientes/CPA-04479-19/2019
A/chicken/Aguascalientes/CPA-03336-19/2019
A/chicken/Mexico/1863/2019
A/chicken/Aguascalientes/CPA-08553-19/2019
A/chicken/Jalisco/CPA-07736-19/2019
A/chicken/Mexico/1864/2019
A/chicken/Jalisco/CPA-07737-19/2019
A/chicken/Aguascalientes/CPA-08545-19/2019
A/chicken/Aguascalientes/CPA-08115-19/2019
A/chicken/Puebla/CPA-03924-19/2019
A/chicken/Mexico/1643/2019
A/chicken/Puebla/CPA-06927-19/2019
A/chicken/Guanajuato/CPA-08992-19/2019
A/chicken/Aguascalientes/CPA-07048-19/2019
A/chicken/Guanajuato/CPA-05981-19/2019
A/chicken/Guanajuato/CPA-07268-19/2019
A/chicken/Guanajuato/CPA-07267-19/2019
A/chicken/Guanajuato/CPA-08990-19/2019
A/chicken/Queretaro/CPA-09543-19/2019
A/chicken/Guanajuato/CPA-08523-19/2019
A/chicken/San-Luis-Potosi/CPA-05885-19/2019
A/chicken/Jalisco/CPA-07139-19/2019
A/chicken/San-Luis-Potosi/CPA-05890-19/2019
A/chicken/Jalisco/CPA-07140-19/2019
A/chicken/Queretaro/CPA-09515-19/2019
A/chicken/Guanajuato/CPA-03879-19/2019
A/chicken/Aguascalientes/CPA-08331-19/2019
A/chicken/Guanajuato/CPA-04382-19/2019
A/chicken/Queretaro/CPA-04112-19/2019
A/chicken/Jalisco/CPA-06506-19/2019
A/chicken/Jalisco/CPA-06504-19/2019
A/chicken/Jalisco/CPA-07195-19/2019
A/chicken/Aguascalientes/CPA-07181-19/2019
A/chicken/Jalisco/CPA-06016-19/2019_2019
A/chicken/Jalisco/CPA-08826-19/2019
A/chicken/Jalisco/CPA-08831-19/2019
A/chicken/Jalisco/CPA-03447-19/2019
A/chicken/Jalisco/CPA-04649-19/2019
A/chicken/Jalisco/CPA-05091-19/2019
A/chicken/Jalisco/CPA-06432-19/2019_2019
A/chicken/Jalisco/CPA-04758-19/2019
A/chicken/Jalisco/CPA-07541-19/2019
A/chicken/Jalisco/CPA-07715-19/2019
A/chicken/Jalisco/CPA-08103-19/2019
A/chicken/Jalisco/CPA-06799-19/2019
A/chicken/Jalisco/CPA-04141-19/2019_2019
A/chicken/Jalisco/CPA-08244-19/2019
A/chicken/Jalisco/CPA-03604-19/2019
A/chicken/Jalisco/CPA-03607-19/2019
A/chicken/Jalisco/CPA-04262-19/2019
A/chicken/Jalisco/CPA-16963-18/2018
A/chicken/Puebla/CPA-01572-18/2018
A/chicken/Hidalgo/CPA-41295-17/2017
A/chicken/Morelos/CPA-05154-17/2017
A/chicken/Puebla/CPA-14731-17/2017
A/chicken/Jalisco/LVVX17186-2/2017
A/chicken/Yucatan/CPA-15038-17/2017
A/chicken/Jalisco/CPA-06811-19/2019
A/chicken/Jalisco/CPA-09057-19/2019
A/chicken/Veracruz/CPA-21377-17/2017
A/chicken/Veracruz/CPA-13920-18/2018
A/chicken/Queretaro/CPA-11747-18/2018
A/chicken/Veracruz/CPA-05599-17/2017
A/chicken/Veracruz/CPA-05600-17/2017
A/chicken/Queretaro/CPA-02178-17/2017
A/chicken/Queretaro/CPA-02179-17/2017
A/chicken/Queretaro/CPA-02177-17/2017
A/chicken/Guanajuato/CPA-17726-17/2017
A/chicken/Queretaro/CPA-06507-17/2017
A/chicken/Veracruz/CPA-04704-17/2017
A/chicken/Veracruz/CPA-00736-17/2017
A/chicken/Veracruz/CPA-00735-17/2017
A/chicken/Jalisco/CPA-49745-16/2016
A/chicken/Jalisco/CPA-03072-17/2017
A/chicken/Hidalgo/CPA-48304-16/2016
A/chicken/Queretaro/CPA-04673-19/2019
A/chicken/Hidalgo/CPA-03739-17/2017
A/chicken/Aguascalientes/CPA-20747-17/2017
A/chicken/Morelos/CPA-18659-17/2017
A/chicken/Jalisco/CPA-00823-17/2017
A/chicken/Jalisco/CPA-00822-17/2017
A/chicken/Jalisco/CPA-06116-17/2017
A/chicken/Jalisco/CPA-03518-17/2017
A/chicken/Jalisco/CPA-49950-16/2016
A/chicken/Michoacan/CPA-49737-16/2016
A/chicken/Michoacan/CPA-48912-16/2016
A/chicken/Michoacan/CPA-48911-16/2016
A/chicken/Jalisco/CPA-22166-17/2017
A/chicken/Guanajuato/CPA-44870-17/2017
A/chicken/Jalisco/CPA-02169-17/2017
A/chicken/Jalisco/CPA-03198-17/2017
A/chicken/Guanajuato/CPA-11248-17/2017
A/chicken/Guanajuato/CPA-00801-17/2017
A/chicken/Jalisco/CPA-12377-17/2017
A/chicken/Jalisco/CPA-49007-16/2016
A/chicken/Guanajuato/CPA-12461-17/2017
A/chicken/Jalisco/LVVX17144-1/2017
A/chicken/Queretaro/CPA-03807-19/2019
A/chicken/Mexico/CPA-19234-17/2017
A/chicken/Queretaro/CPA-03805-19/2019
A/chicken/Queretaro/CPA-03806-19/2019
A/chicken/Morelos/CPA-02447-17/2017
A/chicken/Guanajuato/CPA-15050-17/2017
A/chicken/Queretaro/CPA-05386-17/2017
A/chicken/Aguascalientes/IA13/2010
A/chicken/Durango/CPA-08970-18/2018
A/chicken/Guanajuato/CPA-00114-18/2018
A/chicken/Coahuila/CPA-17850-17/2017
A/chicken/Coahuila/IA20/2011
A/chicken/Colima/CPA-04929-17/2017
A/chicken/Coahuila/1433-15/2006
A/chicken/Durango/1433-8/2005
A/chicken/Durango/1433-4/2005
A/chicken/Hidalgo/1433-6/2005
A/chicken/Durango/1433-9/2006
A/chicken/Mexico/1433-5/2005
A/chicken/Ibaraki/1/2005
A/chicken/Mexico/1433-13/2006
A/chicken/Durango/1433-10/2006
A/chicken/El_Salvador/102711-1/2001
A/chicken/El_Salvador/102711-2/2001
A/chicken/Guatemala/45511-5/2000
A/chicken/Guatemala/45511-3/2000
A/chicken/Guatemala/194573/2002
A/chicken/Guatemala/270475-4/2003
A/chicken/Guatemala/45511-2/2000
A/chicken/Guatemala/45511-1/2000
A/chicken/Guatemala/45511-4/2000
A/chicken/Veracruz/233-7607/1998
A/chicken/Guatemala/22066/1998
A/chicken/Veracruz/232-6169/1998
A/chicken/Veracruz/1433-1/2006
A/chicken/Puebla/14585-622/1994
A/chicken/Puebla/8623-607/1994
A/chicken/Puebla/8624-602/1994
A/chicken/Puebla/14590-658/1994
A/chicken/Puebla/14587-644/1994
A/chicken/Dominican_Republic/19-020695-014_1590-19_7/2019
A/chicken/Dominican_Republic/19-020695-005_705-19_10/2019
A/chicken/Dominican_Republic/19-020695-002_671-19_13/2019
A/chicken/Dominican_Republic/19-020695-004_671-19_15/2019
A/chicken/Dominican_Republic/19-020695-003_671-19_14/2019
A/chicken/Dominican_Republic/19-020695-001_529-19_9/2018
A/chicken/Dominican_Republic/19-020695-010_729-19_2/2019
A/chicken/Dominican_Republic/19-020695-015_1590-19_8/2019
A/chicken/Dominican_Republic/19-020695-013_1136-19_11/2019
A/chicken/Dominican_Republic/19-020695-012_1107-19_16/2019
A/chicken/Dominican_Republic/18-044726-013/2018
A/chicken/Dominican_Republic/17-035772-3/2017
A/chicken/Dominican_Republic/17-035772-2/2017
A/chicken/Dominican_Republic/17-035000-5/2017
A/chicken/Dominican_Republic/17-035000-15/2017
A/chicken/Dominican_Republic/17-041237-004/2017
A/chicken/Dominican_Republic/17-041237-001/2017
A/chicken/Dominican_Republic/19-020695-011_794-19_17/2019
A/chicken/Dominican_Republic/07-002397-7/2007
A/chicken/Hidalgo/26654-1368/1994
A/chicken/Veracruz/28159-398/1995
A/chicken/Mexico/26654-1374/1994
A/chicken/Hidalgo/28159-232/1994
A/chicken/Mexico/31381-6/1994
A/chicken/Mexico/31381-1/1994
A/chicken/Mexico/31381-2/1994
A/chicken/Mexico/31382-1/1994
A/chicken/Mexico/31381-3/1994
A/chicken/Mexico/31381-4/1994
A/chicken/San_Luis_Potosi/7651/1996
A/chicken/Chis/15224/1997
A/chicken/Chiapas/15405/1997
A/chicken/Puebla/28159-474/1995
A/chicken/Chiapas/28159-488/1995
A/chicken/Mexico/31381-5/1994
A/chicken/Queretaro/7653-20/1995
A/chicken/Queretaro/14588-19/1995
A/chicken/Queretaro/22019-853/1996
A/chicken/Jalisco/28159-600/1995
A/chicken/Michoacan/28159-530/1995
A/chicken/Hidalgo/28159-460/1995
A/chicken/Jalisco/14589-660/1994
A/chicken/Guanajuato/28159-331/1995
A/chicken/Jalisco/229-4592/1998
A/chicken/Morelos/227-4353/1998
A/chicken/Guatemala/22189/1998
A/chicken/Chiapas/15224/1997
A/chicken/Mexico/15407/1997
A/chicken/Mexico/31381-7/1994
0.004

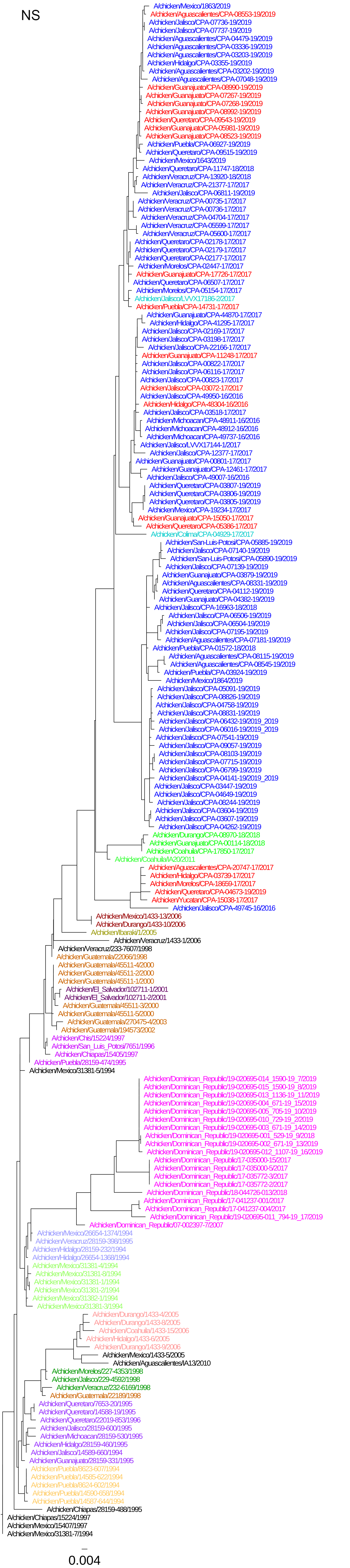
NS
A/chicken/Mexico/1863/2019
A/chicken/Aguascalientes/CPA-08553-19/2019
A/chicken/Jalisco/CPA-07736-19/2019
A/chicken/Jalisco/CPA-07737-19/2019
A/chicken/Aguascalientes/CPA-04479-19/2019
A/chicken/Aguascalientes/CPA-03336-19/2019
A/chicken/Aguascalientes/CPA-03203-19/2019
A/chicken/Hidalgo/CPA-03355-19/2019
A/chicken/Aguascalientes/CPA-03202-19/2019
A/chicken/Aguascalientes/CPA-07048-19/2019
A/chicken/Guanajuato/CPA-08990-19/2019
A/chicken/Guanajuato/CPA-07267-19/2019
A/chicken/Guanajuato/CPA-07268-19/2019
A/chicken/Guanajuato/CPA-08992-19/2019
A/chicken/Queretaro/CPA-09543-19/2019
A/chicken/Guanajuato/CPA-05981-19/2019
A/chicken/Guanajuato/CPA-08523-19/2019
A/chicken/Puebla/CPA-06927-19/2019
A/chicken/Queretaro/CPA-09515-19/2019
A/chicken/Mexico/1643/2019
A/chicken/Queretaro/CPA-11747-18/2018
A/chicken/Veracruz/CPA-13920-18/2018
A/chicken/Veracruz/CPA-21377-17/2017
A/chicken/Jalisco/CPA-06811-19/2019
A/chicken/Veracruz/CPA-00735-17/2017
A/chicken/Veracruz/CPA-00736-17/2017
A/chicken/Veracruz/CPA-04704-17/2017
A/chicken/Veracruz/CPA-05599-17/2017
A/chicken/Veracruz/CPA-05600-17/2017
A/chicken/Queretaro/CPA-02178-17/2017
A/chicken/Queretaro/CPA-02179-17/2017
A/chicken/Queretaro/CPA-02177-17/2017
A/chicken/Morelos/CPA-02447-17/2017
A/chicken/Guanajuato/CPA-17726-17/2017
A/chicken/Queretaro/CPA-06507-17/2017
A/chicken/Morelos/CPA-05154-17/2017
A/chicken/Jalisco/LVVX17186-2/2017
A/chicken/Puebla/CPA-14731-17/2017
A/chicken/Guanajuato/CPA-44870-17/2017
A/chicken/Hidalgo/CPA-41295-17/2017
A/chicken/Jalisco/CPA-02169-17/2017
A/chicken/Jalisco/CPA-03198-17/2017
A/chicken/Jalisco/CPA-22166-17/2017
A/chicken/Guanajuato/CPA-11248-17/2017
A/chicken/Jalisco/CPA-00822-17/2017
A/chicken/Jalisco/CPA-06116-17/2017
A/chicken/Jalisco/CPA-00823-17/2017
A/chicken/Jalisco/CPA-03072-17/2017
A/chicken/Jalisco/CPA-49950-16/2016
A/chicken/Hidalgo/CPA-48304-16/2016
A/chicken/Jalisco/CPA-03518-17/2017
A/chicken/Michoacan/CPA-48911-16/2016
A/chicken/Michoacan/CPA-48912-16/2016
A/chicken/Michoacan/CPA-49737-16/2016
A/chicken/Jalisco/LVVX17144-1/2017
A/chicken/Jalisco/CPA-12377-17/2017
A/chicken/Guanajuato/CPA-00801-17/2017
A/chicken/Guanajuato/CPA-12461-17/2017
A/chicken/Jalisco/CPA-49007-16/2016
A/chicken/Queretaro/CPA-03807-19/2019
A/chicken/Queretaro/CPA-03806-19/2019
A/chicken/Queretaro/CPA-03805-19/2019
A/chicken/Mexico/CPA-19234-17/2017
A/chicken/Guanajuato/CPA-15050-17/2017
A/chicken/Queretaro/CPA-05386-17/2017
A/chicken/Colima/CPA-04929-17/2017
A/chicken/San-Luis-Potosi/CPA-05885-19/2019
A/chicken/Jalisco/CPA-07140-19/2019
A/chicken/San-Luis-Potosi/CPA-05890-19/2019
A/chicken/Jalisco/CPA-07139-19/2019
A/chicken/Guanajuato/CPA-03879-19/2019
A/chicken/Aguascalientes/CPA-08331-19/2019
A/chicken/Queretaro/CPA-04112-19/2019
A/chicken/Guanajuato/CPA-04382-19/2019
A/chicken/Jalisco/CPA-16963-18/2018
A/chicken/Jalisco/CPA-06506-19/2019
A/chicken/Jalisco/CPA-06504-19/2019
A/chicken/Jalisco/CPA-07195-19/2019
A/chicken/Aguascalientes/CPA-07181-19/2019
A/chicken/Puebla/CPA-01572-18/2018
A/chicken/Aguascalientes/CPA-08115-19/2019
A/chicken/Aguascalientes/CPA-08545-19/2019
A/chicken/Puebla/CPA-03924-19/2019
A/chicken/Mexico/1864/2019
A/chicken/Jalisco/CPA-05091-19/2019
A/chicken/Jalisco/CPA-08826-19/2019
A/chicken/Jalisco/CPA-04758-19/2019
A/chicken/Jalisco/CPA-08831-19/2019
A/chicken/Jalisco/CPA-06432-19/2019_2019
A/chicken/Jalisco/CPA-06016-19/2019_2019
A/chicken/Jalisco/CPA-07541-19/2019
A/chicken/Jalisco/CPA-09057-19/2019
A/chicken/Jalisco/CPA-08103-19/2019
A/chicken/Jalisco/CPA-07715-19/2019
A/chicken/Jalisco/CPA-06799-19/2019
A/chicken/Jalisco/CPA-04141-19/2019_2019
A/chicken/Jalisco/CPA-03447-19/2019
A/chicken/Jalisco/CPA-04649-19/2019
A/chicken/Jalisco/CPA-08244-19/2019
A/chicken/Jalisco/CPA-03604-19/2019
A/chicken/Jalisco/CPA-03607-19/2019
A/chicken/Jalisco/CPA-04262-19/2019
A/chicken/Durango/CPA-08970-18/2018
A/chicken/Guanajuato/CPA-00114-18/2018
A/chicken/Coahuila/CPA-17850-17/2017
A/chicken/Coahuila/IA20/2011
A/chicken/Aguascalientes/CPA-20747-17/2017
A/chicken/Hidalgo/CPA-03739-17/2017
A/chicken/Morelos/CPA-18659-17/2017
A/chicken/Queretaro/CPA-04673-19/2019
A/chicken/Yucatan/CPA-15038-17/2017
A/chicken/Jalisco/CPA-49745-16/2016
A/chicken/Mexico/1433-13/2006
A/chicken/Durango/1433-10/2006
A/chicken/Ibaraki/1/2005
A/chicken/Veracruz/1433-1/2006
A/chicken/Veracruz/233-7607/1998
A/chicken/Guatemala/22066/1998
A/chicken/Guatemala/45511-4/2000
A/chicken/Guatemala/45511-2/2000
A/chicken/Guatemala/45511-1/2000
A/chicken/El_Salvador/102711-1/2001
A/chicken/El_Salvador/102711-2/2001
A/chicken/Guatemala/45511-3/2000
A/chicken/Guatemala/45511-5/2000
A/chicken/Guatemala/270475-4/2003
A/chicken/Guatemala/194573/2002
A/chicken/Chis/15224/1997
A/chicken/San_Luis_Potosi/7651/1996
A/chicken/Chiapas/15405/1997
A/chicken/Puebla/28159-474/1995
A/chicken/Mexico/31381-5/1994
A/chicken/Dominican_Republic/19-020695-014_1590-19_7/2019
A/chicken/Dominican_Republic/19-020695-015_1590-19_8/2019
A/chicken/Dominican_Republic/19-020695-013_1136-19_11/2019
A/chicken/Dominican_Republic/19-020695-004_671-19_15/2019
A/chicken/Dominican_Republic/19-020695-005_705-19_10/2019
A/chicken/Dominican_Republic/19-020695-010_729-19_2/2019
A/chicken/Dominican_Republic/19-020695-003_671-19_14/2019
A/chicken/Dominican_Republic/19-020695-001_529-19_9/2018
A/chicken/Dominican_Republic/19-020695-002_671-19_13/2019
A/chicken/Dominican_Republic/19-020695-012_1107-19_16/2019
A/chicken/Dominican_Republic/17-035000-15/2017
A/chicken/Dominican_Republic/17-035000-5/2017
A/chicken/Dominican_Republic/17-035772-3/2017
A/chicken/Dominican_Republic/17-035772-2/2017
A/chicken/Dominican_Republic/18-044726-013/2018
A/chicken/Dominican_Republic/17-041237-001/2017
A/chicken/Dominican_Republic/17-041237-004/2017
A/chicken/Dominican_Republic/19-020695-011_794-19_17/2019
A/chicken/Dominican_Republic/07-002397-7/2007
A/chicken/Mexico/26654-1374/1994
A/chicken/Veracruz/28159-398/1995
A/chicken/Hidalgo/28159-232/1994
A/chicken/Hidalgo/26654-1368/1994
A/chicken/Mexico/31381-4/1994
A/chicken/Mexico/31381-8/1994
A/chicken/Mexico/31381-1/1994
A/chicken/Mexico/31381-2/1994
A/chicken/Mexico/31382-1/1994
A/chicken/Mexico/31381-3/1994
A/chicken/Durango/1433-4/2005
A/chicken/Durango/1433-8/2005
A/chicken/Coahuila/1433-15/2006
A/chicken/Hidalgo/1433-6/2005
A/chicken/Durango/1433-9/2006
A/chicken/Mexico/1433-5/2005
A/chicken/Aguascalientes/IA13/2010
A/chicken/Morelos/227-4353/1998
A/chicken/Jalisco/229-4592/1998
A/chicken/Veracruz/232-6169/1998
A/chicken/Guatemala/22189/1998
A/chicken/Queretaro/7653-20/1995
A/chicken/Queretaro/14588-19/1995
A/chicken/Queretaro/22019-853/1996
A/chicken/Jalisco/28159-600/1995
A/chicken/Michoacan/28159-530/1995
A/chicken/Hidalgo/28159-460/1995
A/chicken/Jalisco/14589-660/1994
A/chicken/Guanajuato/28159-331/1995
A/chicken/Puebla/8623-607/1994
A/chicken/Puebla/14585-622/1994
A/chicken/Puebla/8624-602/1994
A/chicken/Puebla/14590-658/1994
A/chicken/Puebla/14587-644/1994
A/chicken/Chiapas/28159-488/1995
A/chicken/Chiapas/15224/1997
A/chicken/Mexico/15407/1997
A/chicken/Mexico/31381-7/1994
0.004
