# Supplementary material for: Evolutionary Dynamics of Mexican Lineage H5N2 Avian Influenza Viruses
Source: Viruses. 2022 May 3;14(5):958. doi: 10.3390/v14050958 (PMC9146523; doi:10.3390/v14050958)
Supplement: Supplementary file 1 [file viruses-14-00958-s001.zip › Figure S3.pdf]

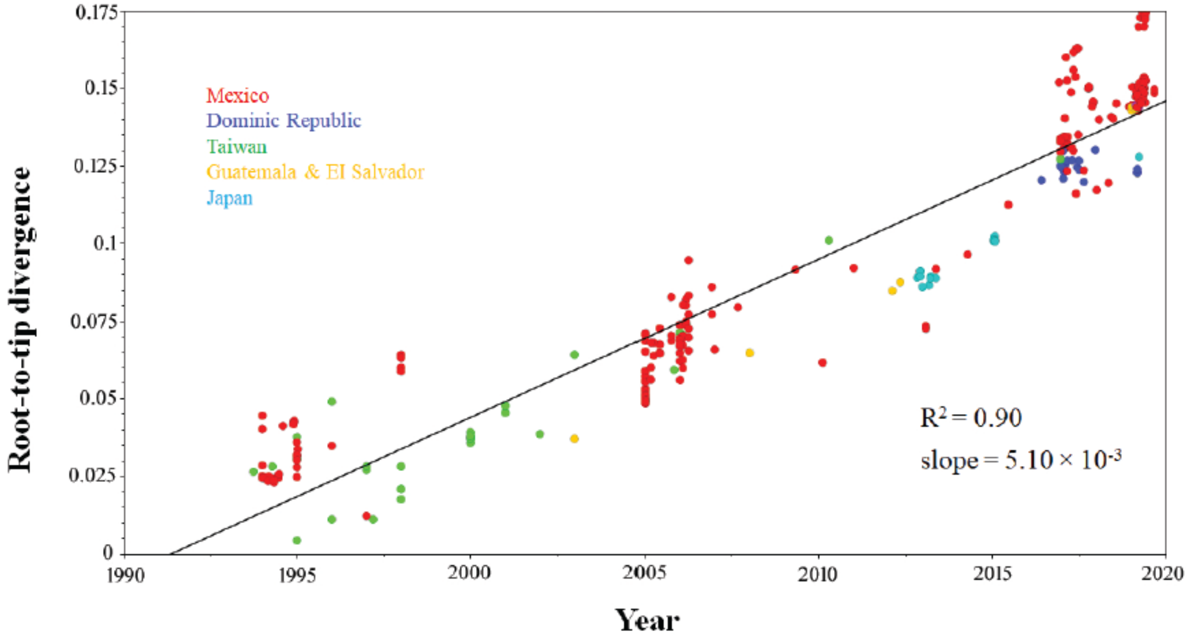

Figure S3. Clock-like nature of Mexican lineage H5N2 AIV evolution. Regression of root-to-tip genetic divergence against sampling date was performed based on a phylogeny of 284 complete HA sequences. Sequences are colored according to their geographic locations. Red, Mexico; blue, Dominic Republic; green, Taiwan; yellow, Guatemala and EI Salvador; cyan, Japan.
