# Supplementary material for: Evolutionary Dynamics of Mexican Lineage H5N2 Avian Influenza Viruses
Source: Viruses. 2022 May 3;14(5):958. doi: 10.3390/v14050958 (PMC9146523; doi:10.3390/v14050958)
Supplement: Supplementary file 1 [file viruses-14-00958-s001.zip › Figure S4.pdf]

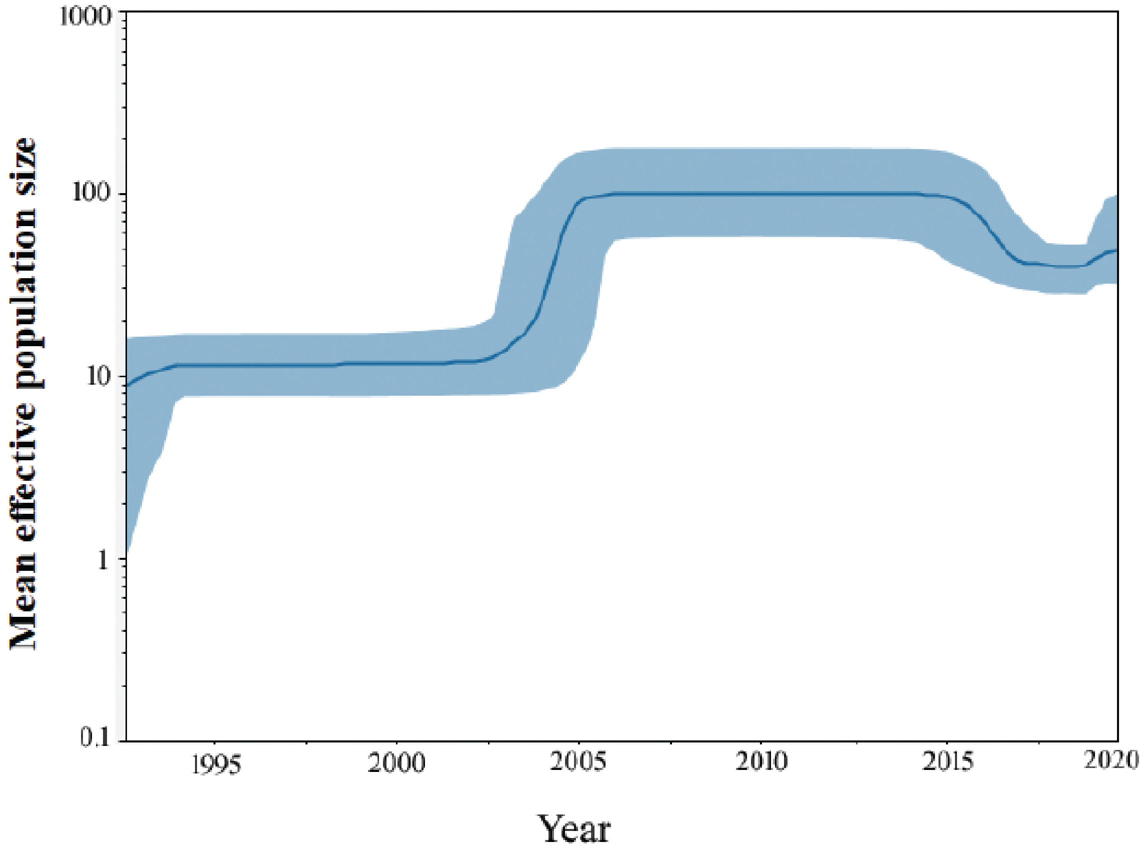


**Figure S4**. Bayesian skyline plot showing the demographic history of Mexican lineage H5N2 AIV circulating globally. The H5 HA sequences from 284 H5N2 viruses were included in the analysis. The thick colored line is the mean estimate, and the 95% HPD is indicated by the shaded area.
