# Supplementary material for: Evolutionary Dynamics of Mexican Lineage H5N2 Avian Influenza Viruses
Source: Viruses. 2022 May 3;14(5):958. doi: 10.3390/v14050958 (PMC9146523; doi:10.3390/v14050958)
Supplement: Supplementary file 1 [file viruses-14-00958-s001.zip › Figure S5.pdf]

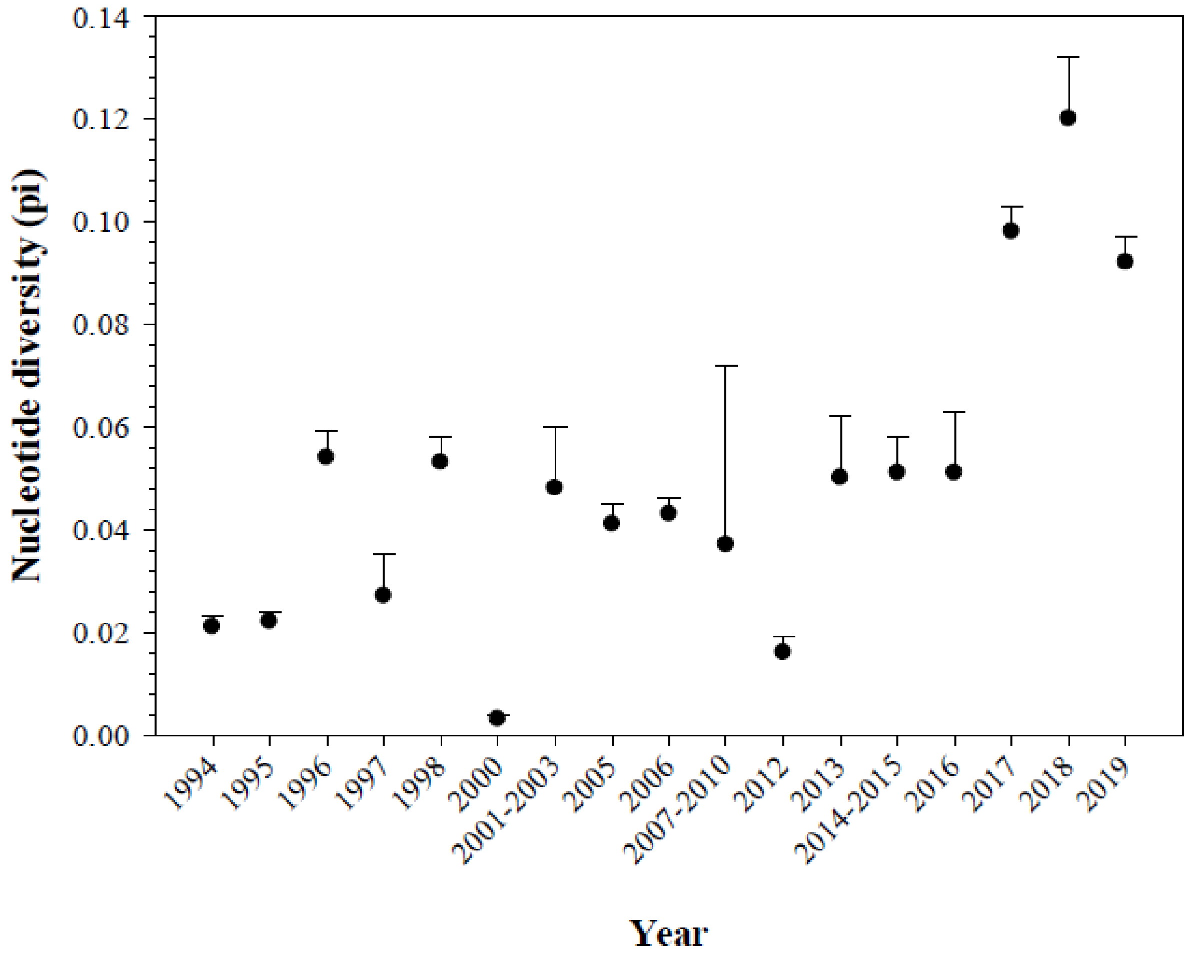

Figure S5. Temporal changes in genetic diversities of Mexican lineage H5N2 AIVs. The HA genes from 284 H5N2 viruses were used to calculate the genetic diversity (π) detected in each year.
