# Supplementary material for: Evolutionary Dynamics of Mexican Lineage H5N2 Avian Influenza Viruses
Source: Viruses. 2022 May 3;14(5):958. doi: 10.3390/v14050958 (PMC9146523; doi:10.3390/v14050958)
Supplement: Supplementary file 1 [file viruses-14-00958-s001.zip › Table S1.pdf]

Supplementary Table S1. 2016-2019 Mexican H5N2 AIVs used in this study.

Strain	Collection date (d/m/y)	Type of surveillance	Type of sampled bird	GenBank accession no.	HA cleavage site motif
A/chicken/Hidalgo/CPA-48304-16/2016	05-12-2016	Active surveillance	Healthy	MZ564905-MZ564912	PQRKTR/G
A/chicken/Michoacan/CPA-48911-16/2016	13-12-2016	Active surveillance	Healthy	MZ565233-MZ565240	PQRKTR/G
A/chicken/Michoacan/CPA-48912-16/2016	13-12-2016	Active surveillance	Healthy	MZ565241-MZ565248	PQRKTR/G
A/chicken/Jalisco/CPA-49007-16/2016	13-12-2016	Active surveillance	Healthy	MZ565201-MZ565208	PQRKTR/G
A/chicken/Michoacan/CPA-49737-16/2016	19-12-2016	Active surveillance	Healthy	MZ565249-MZ565256	PQKRKR/G
A/chicken/Jalisco/CPA-49745-16/2016	19-12-2016	Active surveillance	Healthy	MZ565209-MZ565216	PQRKTR/G
A/chicken/Jalisco/CPA-49950-16/2016	20-12-2016	Active surveillance	Healthy	MZ565217-MZ565224	PQRKTR/G
A/chicken/Jalisco/CPA-00822-17/2017	13-01-2017	Active surveillance	Healthy	MZ564913-MZ564920	PQRKTR/G
A/chicken/Jalisco/CPA-00823-17/2017	13-01-2017	Active surveillance	Healthy	MZ564921-MZ564928	PQRKTR/G
A/chicken/Veracruz/CPA-00735-17/2017	13-01-2017	Active surveillance	Healthy	MZ565433-MZ565440	PQRKTR/G
A/chicken/Veracruz/CPA-00736-17/2017	13-01-2017	Active surveillance	Healthy	MZ565441-MZ565448	PQRKTR/G
A/chicken/Guanajuato/CPA-00801-17/2017	16-01-2017	Active surveillance	Healthy	MZ564769-MZ564776	PQRKTR/G
A/chicken/Jalisco/CPA-02169-17/2017	25-01-2017	Active surveillance	Healthy	MZ564929-MZ564936	PQRKTR/G
A/chicken/Queretaro/CPA-02177-17/2017	31-01-2017	Active surveillance	Dead corpse	MZ565313-MZ565320	PQRKTR/G
A/chicken//Queretaro/CPA-02178-17/2017	31-01-2017	Active surveillance	Dead corpse	MZ565321-MZ565328	PQKRKR/G
A/chicken/Queretaro/CPA-02179-17/2017	31-01-2017	Active surveillance	Dead corpse	MZ565329-MZ565336	PQRKTR/G
A/chicken/Morelos/CPA-02447-17/2017	31-01-2017	Active surveillance	Sentinel	MZ565257-MZ565264	PQRKTR/G
A/chicken/Jalisco/CPA-03072-17/2017	02-02-2017	Passive surveillance	Sick	MZ564937-MZ564944	PQRKTR/G
A/chicken/Jalisco/CPA-03198-17/2017	03-02-2017	Active surveillance	Healthy	MZ564945-MZ564952	PQRKTR/G
A/chicken/Jalisco/CPA-03518-17/2017	08-02-2017	Active surveillance	Healthy	MZ564961-MZ564968	PQRKTR/G
A/chicken/Hidalgo/CPA-03739-17/2017	13-02-2017	Active surveillance	Healthy	MZ564889-MZ564896	PQRKTR/G
A/chicken/Veracruz/CPA-04704-17/2017	22-02-2017	Active surveillance	Vaccinated	MZ565449-MZ565456	PQRKTR/G
A/chicken/Morelos/CPA-05154-17/2017	23-02-2017	Active surveillance	Sentinel	MZ565265-MZ565272	PQRKTR/G
A/chicken/Colima/CPA-04929-17/2017	24-02-2017	Active surveillance	Healthy	MZ564745-MZ564752	PQRKTR/G
A/chicken/Queretaro/CPA-05386-17/2017	24-02-2017	Active surveillance	Healthy	MZ565377-MZ565384	PQRKTR/G
A/chicken/Jalisco/CPA-06116-17/2017	01-03-2017	Active surveillance	Healthy	MZ565033-MZ565040	PQKRKR/G
A/chicken/Veracruz/CPA-05599-17/2017	02-03-2017	Active surveillance	Vaccinated	MZ565457-MZ565464	PQRKTR/G
A/chicken/Veracruz/CPA-05600-17/2017	02-03-2017	Active surveillance	Vaccinated	MZ565465-MZ565472	PQKRKR/G
A/chicken/Queretaro/CPA-06507-17/2017	06-03-2017	Active surveillance	Healthy	MZ565385-MZ565392	PQRKTR/G
A/chicken/Guanajuato/CPA-11248-17/2017	07-04-2017	Active surveillance	Sentinel	MZ564841-MZ564848	PQRKTR/G
A/chicken/Guanajuato/CPA-12461-17/2017	18-04-2017	Active surveillance	Healthy	MZ564849-MZ564856	PQRKTR/G
A/chicken/Jalisco/CPA-12377-17/2017	19-04-2017	Active surveillance	Healthy	MZ565177-MZ565184	PQRKTR/G
A/chicken/Puebla/CPA-14731-17/2017	04-05-2017	Active surveillance	Vaccinated	MZ565305-MZ565312	PQRKTR/G
A/chicken/Guanajuato/CPA-15050-17/2017	05-05-2017	Active surveillance	Dead corpse	MZ564857-MZ564864	PQRKTR/G
A/chicken/Yucatan/CPA-15038-17/2017	06-05-2017	Passive surveillance	Sick	MZ565489-MZ565496	LQRKTR/G
A/chicken/Guanajuato/CPA-17726-17/2017	25-05-2017	Active surveillance	Sentinel	MZ564865-MZ564872	PQRKTR/G
A/chicken/Coahuila/CPA-17850-17/2017	01-06-2017	Active surveillance	Vaccinated	MZ564737-MZ564744	PQRKTR/G
A/chicken/Morelos/CPA-18659-17/2017	06-06-2017	Active surveillance	Sentinel	MZ565273-MZ565280	PQRKTR/G
A/chicken/Mexico/CPA-19234-17/2017	13-06-2017	Active surveillance	Dead corpse	MZ565225-MZ565232	PQKRKR/G
A/chicken/Aguascalientes/CPA-20747-17/2017	22-06-2017	Active surveillance	Sentinel	MZ564721-MZ564728	PQRKTR/G
A/chicken/Veracruz/CPA-21377-17/2017	23-06-2017	Active surveillance	Vaccinated	MZ565481-MZ565488	PQRKTR/G
A/chicken/Jalisco/CPA-22166-17/2017	29-06-2017	Active surveillance	Healthy	MZ565193-MZ565200	PQKRKR/G

A/chicken/Hidalgo/CPA-41295-17/2017	17-11-2017	Active surveillance	Healthy	MZ564897-MZ564904	PQRKTR/G
A/chicken/Guanajuato/CPA-44870-17/2017	19-12-2017	Active surveillance	Sentinel	MZ564873-MZ564880	PQRKTR/G
A/chicken/Guanajuato/CPA-00114-18/2018	03-01-2018	Active surveillance	Sentinel	MZ564761-MZ564768	PQRKTR/G
A/chicken/Puebla/CPA-01572-18/2018	28-01-2018	Active surveillance	Vaccinated	MZ565281-MZ565288	PQRKTR/G
A/chicken/Durango/CPA-08970-18/2018	08-05-2018	Active surveillance	Sentinel	MZ564753-MZ564760	PQRKTR/G
A/chicken/Queretaro/CPA-11747-18/2018	06-06-2018	Active surveillance	Healthy	MZ565409-MZ565416	PQRKTR/G
A/chicken/Veracruz/CPA-13920-18/2018	28-06-2018	Active surveillance	Healthy	MZ565473-MZ565480	PQRKTR/G
A/chicken/Jalisco/CPA-16963-18/2018	31-07-2018	Active surveillance	Healthy	MZ565185-MZ565192	PQRKTR/G
A/chicken/Aguascalientes/CPA-03203-19/2019	25-02-2019	Active surveillance	Sentinel	MZ564657-MZ564664	PQKRKR/G
A/chicken/Aguascalientes/CPA-03202-19/2019	25-02-2019	Active surveillance	Sentinel	MZ564649-MZ564656	PQKRKR/G
A/chicken/Hidalgo/CPA-03355-19/2019	27-02-2019	Active surveillance	Healthy	MZ564881-MZ564888	PQRKTR/G
A/chicken/Aguascalientes/CPA-03336-19/2019	27-02-2019	Active surveillance	Sentinel	MZ564665-MZ564672	PQRKTR/G
A/chicken/Jalisco/CPA-03447-19/2019	28-02-2019	Active surveillance	Healthy	MZ564953-MZ564960	PQRKTR/G
A/chicken/Jalisco/CPA-03607-19/2019	05-03-2019	Active surveillance	Healthy	MZ564977-MZ564984	PQRKTR/G
A/chicken/Jalisco/CPA-03604-19/2019	05-03-2019	Active surveillance	Healthy	MZ564969-MZ564976	PQRKTR/G
A/chicken/Queretaro/CPA-03806-19/2019	07-03-2019	Active surveillance	Healthy	MZ565345-MZ565352	PQRKTR/G
A/chicken/Queretaro/CPA-03807-19/2019	07-03-2019	Active surveillance	Sentinel	MZ565353-MZ565360	PQRKTR/G
A/chicken/Queretaro/CPA-03805-19/2019	07-03-2019	Active surveillance	Sentinel	MZ565337-MZ565344	PQRKTR/G
A/chicken/Guanajuato/CPA-03879-19/2019	08-03-2019	Active surveillance	Sentinel	MZ564777-MZ564784	PQRKTR/G
A/chicken/Puebla/CPA-03924-19/2019	11-03-2019	Active surveillance	Healthy	MZ565289-MZ565296	PQRKTR/G
A/chicken/Queretaro/CPA-04112-19/2019	12-03-2019	Active surveillance	Healthy	MZ565361-MZ565368	PQRKTR/G
A/chicken/Jalisco/CPA-04141-19/2019	13-03-2019	Active surveillance	Healthy	MZ564985-MZ564992	PQRKTR/G
A/chicken/Jalisco/CPA-04262-19/2019	14-03-2019	Active surveillance	Healthy	MZ564993-MZ565000	PQRKTR/G
A/chicken/Guanajuato/CPA-04382-19/2019	15-03-2019	Active surveillance	Sentinel	MZ564785-MZ564792	PQRKTR/G
A/chicken/Aguascalientes/CPA-04479-19/2019	19-03-2019	Active surveillance	Sentinel	MZ564673-MZ564680	PQKRKR/G
A/chicken/Jalisco/CPA-04649-19/2019	20-03-2019	Active surveillance	Healthy	MZ565001-MZ565008	PQRKTR/G
A/chicken/Queretaro/CPA-04673-19/2019	20-03-2019	Passive surveillance	Sick	MZ565369-MZ565376	PQRKTR/G
A/chicken/Jalisco/CPA-04758-19/2019	21-03-2019	Active surveillance	Healthy	MZ565009-MZ565016	PQRKTR/G
A/chicken/Jalisco/CPA-05091-19/2019	26-03-2019	Active surveillance	Healthy	MZ565017-MZ565024	PQRKTR/G
A/chicken/Guanajuato/CPA-05981-19/2019	08-04-2019	Active surveillance	Sentinel	MZ564793-MZ564800	PQRKTR/G
A/chicken/San Luis Potosi/CPA-05885-19/2019	08-04-2019	Active surveillance	Sentinel	MZ565417-MZ565424	PQRKTR/G
A/chicken/San Luis Potosi/CPA-05890-19/2019	08-04-2019	Active surveillance	Sentinel	MZ565425-MZ565432	PQRKTR/G
A/chicken/Jalisco/CPA-06016-19/2019	09-04-2019	Active surveillance	Healthy	MZ565025-MZ565032	PQRKTR/G
A/chicken/Jalisco/CPA-06432-19/2019	15-04-2019	Active surveillance	Healthy	MZ565041-MZ565048	PQRKTR/G
A/chicken/Jalisco/CPA-06506-19/2019	15-04-2019	Active surveillance	Healthy	MZ565057-MZ565064	PQRKTR/G
A/chicken/Jalisco/CPA-06504-19/2019	15-04-2019	Active surveillance	Healthy	MZ565049-MZ565056	PQRKTR/G
A/chicken/Jalisco/CPA-06811-19/2019	23-04-2019	Active surveillance	Healthy	MZ565073-MZ565080	PQRKTR/G
A/chicken/Jalisco/CPA-06799-19/2019	23-04-2019	Active surveillance	Healthy	MZ565065-MZ565072	PQKRKR/G
A/chicken/Puebla/CPA-06927-19/2019	24-04-2019	Active surveillance	Vaccinated	MZ565297-MZ565304	PQRKTR/G
A/chicken/Aguascalientes/CPA-07048-19/2019	25-04-2019	Active surveillance	Sentinel	MZ564681-MZ564688	PQKRKR/G
A/chicken/Jalisco/CPA-07140-19/2019	26-04-2019	Active surveillance	Healthy	MZ565089-MZ565096	PQRKTR/G
A/chicken/Jalisco/CPA-07139-19/2019	26-04-2019	Active surveillance	Healthy	MZ565081-MZ565088	PQRETR/G
A/chicken/Guanajuato/CPA-07267-19/2019	29-04-2019	Active surveillance	Sentinel	MZ564801-MZ564808	PQRKTR/G
A/chicken/Jalisco/CPA-07195-19/2019	29-04-2019	Active surveillance	Healthy	MZ565097-MZ565104	PQKRKR/G

A/chicken/Guanajuato/CPA-07268-19/2019	29-04-2019	Active surveillance	Sentinel	MZ564809-MZ564816	PQRKTR/G
A/chicken/Aguascalientes/CPA-07181-19/2019	29-04-2019	Active surveillance	Sentinel	MZ564689-MZ564696	PQRKTR/G
A/chicken/Jalisco/CPA-07541-19/2019	02-05-2019	Active surveillance	Healthy	MZ565105-MZ565112	PQRKTR/G
A/chicken/Jalisco/CPA-07737-19/2019	07-05-2019	Active surveillance	Healthy	MZ565129-MZ565136	PQRKTR/G
A/chicken/Jalisco/CPA-07715-19/2019	07-05-2019	Active surveillance	Healthy	MZ565113-MZ565120	PQKRKR/G
A/chicken/Jalisco/CPA-07736-19/2019	07-05-2019	Active surveillance	Healthy	MZ565121-MZ565128	PQRKTR/G
A/chicken/Aguascalientes/CPA-08115-19/2019	13-05-2019	Active surveillance	Dead corpse	MZ564697-MZ564704	PQRKTR/G
A/chicken/Jalisco/CPA-08103-19/2019	13-05-2019	Active surveillance	Healthy	MZ565137-MZ565144	PQRKTR/G
A/chicken/Jalisco/CPA-08244-19/2019	14-05-2019	Active surveillance	Healthy	MZ565145-MZ565152	SQKRKR/G
A/chicken/Aguascallientes/CPA-08331-19/2019	15-05-2019	Active surveillance	Dead corpse	MZ564729-MZ564736	PQKGKR/G
A/chicken/Guanajuato/CPA-08523-19/2019	20-05-2019	Active surveillance	Sentinel	MZ564817-MZ564824	PQKRKR/G
A/chicken/Aguascalientes/CPA-08545-19/2019	20-05-2019	Active surveillance	Dead corpse	MZ564705-MZ564712	PQRKTR/G
A/chicken/Aguascalientes/CPA-08553-19/2019	20-05-2019	Active surveillance	Sentinel	MZ564713-MZ564720	PQKRKR/G
A/chicken/Jalisco/CPA-08826-19/2019	23-05-2019	Active surveillance	Healthy	MZ565153-MZ565160	PQRKTR/G
A/chicken/Jalisco/CPA-08831-19/2019	23-05-2019	Active surveillance	Healthy	MZ565161-MZ565168	PQKRKR/G
A/chicken/Guanajuato/CPA-08992-19/2019	27-05-2019	Active surveillance	Sentinel	MZ564833-MZ564840	PQRKTR/G
A/chicken/Guanajuato/CPA-08990-19/2019	27-05-2019	Active surveillance	Sentinel	MZ564825-MZ564832	PQRKTR/G
A/chicken/Jalisco/CPA-09057-19/2019	28-05-2019	Active surveillance	Healthy	MZ565169-MZ565176	PQRKTR/G
A/chicken/Queretaro/CPA-09515-19/2019	03-06-2019	Active surveillance	Dead corpse	MZ565393-MZ565400	PQRKTR/G
A/chicken//Queretaro/CPA-09543-19/2019	03-06-2019	Active surveillance	Vaccinated	MZ565401-MZ565408	PQRKTR/G
